# Inhibition of Cxcr4 chemokine receptor signaling improves habituation learning in a zebrafish model of neurofibromatosis

**DOI:** 10.1242/dmm.052509

**Published:** 2026-03-12

**Authors:** Andrew H. Miller, Yeng Yang, Natalie Schmidt, Jaffna Mathiaparanam, Mark E. Berres, Mary C. Halloran

**Affiliations:** ^1^ Department of Integrative Biology, University of Wisconsin-Madison, Madison, WI 53706, USA; ^2^ Department of Neuroscience, University of Wisconsin-Madison, Madison, WI 53706, USA; ^3^ Neuroscience Training Program, University of Wisconsin-Madison, Madison, WI 53706, USA; ^4^ Biotechnology Center, University of Wisconsin-Madison, Madison, WI 53706, USA

**Keywords:** Neurofibromatosis, Habituation, cxcr4, cAMP, Zebrafish

## Abstract

Neurofibromatosis type 1 (NF1) is a neurogenetic disorder caused by loss-of-function mutations in the gene neurofibromin 1 (*NF1*). *NF1* encodes neurofibromin, a multifunctional tumor-suppressing protein that regulates Ras, cAMP and dopamine signaling. NF1 predisposes patients to a wide range of symptoms, including peripheral nerve tumors, brain tumors and cognitive dysfunction. Despite considerable work using animal models to investigate the role of neurofibromin in behavior, translating research into treatment for NF1-associated cognitive dysfunction has not yet been successful. Here, we provide evidence that Cxcr4 chemokine receptor signaling is a regulator of habituation learning and modulator of cAMP-PKA signaling in *nf1* mutant larval zebrafish. Combining a small-molecule drug screen and RNAseq analysis, we show that *cxcr4b* expression is increased in *nf1* mutants and that pharmacological inhibition of Cxcr4 with AMD3100 (plerixafor) improves habituation learning. We further demonstrate that plerixafor activates cAMP-PKA pathway signaling but has limited effects on Ras-Raf-MEK-ERK pathway signaling in the *nf1* mutant brain. CXCR4 has previously been identified as a potential therapeutic target for neurofibromin-deficient tumorigenesis. Our results suggest that Cxcr4 signaling also regulates neurofibromin-dependent cognitive function.

## Teaser

**Editor's choice:** Treatments for neurofibromatosis type 1-associated cognitive dysfunction are needed. Inhibition of chemokine receptor signaling improves habituation learning and increases cAMP signaling in a zebrafish model of neurofibromatosis.

## INTRODUCTION

Neurofibromatosis type 1 (NF1) is an autosomal dominant, neurogenetic disorder caused by loss-of-function mutations in the gene neurofibromin 1 (*NF1*). NF1 is a comparatively common single-gene disorder estimated to affect 1 in 2000-3000 live births (Evans et al., 2010; Huson et al., 1989; Uusitalo et al., 2015). NF1 is associated with a wide range of symptoms involving multiple organ systems. Neurological features, including peripheral nerve tumors, brain tumors and cognitive dysfunction are major clinical concerns (Cimino and Gutmann, 2018). Up to 80% of children with NF1 experience cognitive or behavioral deficits that impact social and academic development (Hyman et al., 2005; Torres Nupan et al., 2017). Despite increased attention on the role of cognitive function in quality of life for patients with NF1 (Krab et al., 2009; Varni et al., 2019), the molecular basis of NF1-associated cognitive dysfunction remains unclear (Miller and Halloran, 2022).

*NF1* encodes neurofibromin, a multifunctional GTPase-activating protein (GAP) that inhibits Ras signaling (Basu et al., 1992; Dasgupta et al., 2005; DeClue et al., 1991; Endo et al., 2013; Johannessen et al., 2005; Marchuk et al., 1991) and activates cAMP signaling (Anastasaki and Gutmann, 2014; Guo et al., 1997; Hannan et al., 2006). cAMP is a second messenger regulated by G protein-coupled receptor (GPCR) modulation of adenylyl cyclase (AC) activity. Dysregulated GPCR signaling is seen in neurofibromin-deficient human and mouse tissue, including increased CXCR4 chemokine receptor signaling associated with tumorigenesis (Karaosmanoglu et al., 2018; Mo et al., 2013; Warrington et al., 2007) and reduced dopamine signaling associated with cognitive function (Brown et al., 2010a, 2011; Diggs-Andrews et al., 2013). Ras and cAMP signaling also have been implicated in the regulation of neurofibromin-dependent cognitive function. Studies in both fruit flies and zebrafish show that loss of neurofibromin results in learning deficits that are rescued by enhancing the cAMP signaling pathway, whereas memory deficits are rescued by inhibiting the Ras signaling pathway (Ho et al., 2007; Wolman et al., 2014). Therefore, the role of neurofibromin might differ in neural circuits controlling distinct cognitive functions. The mechanism by which neurofibromin regulates cAMP signaling is unclear, as Ras-independent (Brown et al., 2010b, 2012; Guo et al., 1997), Ras-dependent (Anastasaki and Gutmann, 2014; Walker et al., 2006), and both Ras-independent and dependent pathways (Hannan et al., 2006) have been proposed.

Translation of research from animal models into treatments for NF1-associated cognitive dysfunction has seen limited success. Clinical trials for patients with NF1 using lovastatin or simvastatin to reduce Ras signaling have demonstrated limited effectiveness in improving learning or other measures of cognitive function (Bearden et al., 2016; Krab et al., 2008; Mainberger et al., 2013; Payne et al., 2016; Stivaros et al., 2018; van der Vaart et al., 2013). In a more recent clinical trial, regulation of Ras signaling through mitogen-activated protein kinase kinase (MEK) inhibitor (MEKi) treatment showed stable but not improved group-level performance on working memory and visual learning/memory tasks (Walsh et al., 2021). Identifying additional drug targets that improve learning in *Nf1* mutant animals and characterizing their mechanisms of action will provide new opportunities to develop treatments for cognitive dysfunction in patients with NF1.

In this study, using a zebrafish model of NF1 (Shin et al., 2012), we took two approaches to gain insight into the molecular mechanisms by which neurofibromin deficiency leads to behavioral and cognitive deficits. The first, a small-molecule drug screen to identify regulators of habituation learning, revealed AMD3100 (hereafter referred to as plerixafor), a modulator of the Cxcl12-Cxcr4 and Cxcl12-Ackr3 chemokine signaling axes, improves habituation deficits in *nf1* mutants. The second, RNAseq analysis to identify changes in gene expression with loss of neurofibromin, showed that chemokine receptor *cxcr4b* is upregulated in *nf1* mutants. Cxcr4 signaling is known to activate Ras-Raf-MEK-ERK signaling and inhibit cAMP-PKA signaling (Busillo and Benovic, 2007). Therefore, we further investigated the effects of pharmacological inhibition of Cxcr4 on these signaling pathways. Using a whole-brain mitogen-activated protein kinase mapping (MAP-mapping) approach, we demonstrated that *nf1* mutant larvae have region-specific increases and decreases in phosphorylated-ERK1/2 (pERK). However, plerixafor treatment produced limited changes in brain pERK levels in wild-type or *nf1* mutant larvae. In contrast, using an enzyme-linked immunosorbent assay (ELISA) and whole-brain immunolabeling, we showed that *nf1* mutant larvae have decreased whole-body cAMP and whole-brain phosphorylated-PKA-substrate (pPKA-substrate) levels that are increased by plerixafor treatment in *nf1* heterozygous mutant larvae. Overall, we have identified Cxcr4 signaling as a potential regulator of neurofibromin-dependent habituation learning and a modulator of cAMP-PKA signaling in *nf1* mutant larval zebrafish.

## RESULTS

### Small-molecule screening reveals regulators of acoustically evoked behaviors in *nfl* mutant larvae

A zebrafish model of NF1 harboring null alleles in the *NF1* orthologs *nf1a* and *nf1b* has previously been developed using a zinc finger nuclease strategy (Shin et al., 2012). We used these *nf1* mutants in an unbiased small-molecule screen to identify potential drug targets that regulate neurofibromin-dependent behaviors. Previous work has shown that zebrafish *nf1* mutants have defects in prepulse inhibition (PPI) and habituation of the acoustic startle response, two measures of sensory filtering that demonstrate the ability of zebrafish larvae to adapt their behavior based on previous sensory experience (Randlett et al., 2019; Shin et al., 2012; Wolman et al., 2014). We measured the effects pharmacological manipulation of cellular signaling has on PPI and habituation learning in *nf1* mutant larvae.

**Fig. 1.**
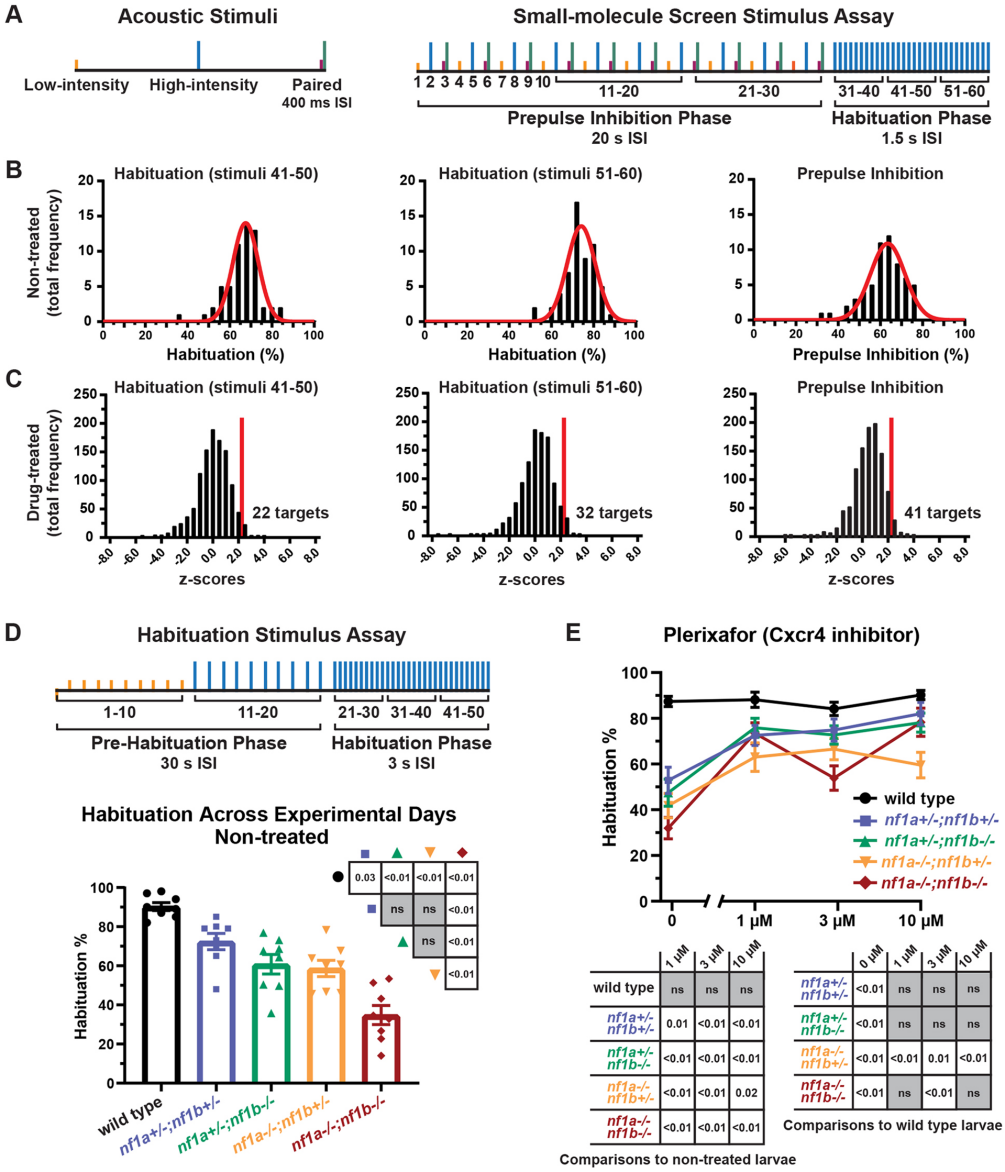
**Small-molecule screen reveals Cxcr4 signaling regulates habituation in *nf1* mutant larvae.** (A) Depiction of the stimulus assay used in combination with small-molecule screen. The low-intensity stimulus was designed to elicit 25% SLC responses. The high-intensity stimulus was designed to elicit 75% SLC responses. Paired low and high intensity stimuli were used during the prepulse inhibition (PPI) phase. The interstimulus interval (ISI) was reduced from 20 s to 1.5 s during the habituation phase. (B) Histograms demonstrating the frequency distribution of habituation and PPI percentage averaged across experimental days in 5 dpf non-treated *nf1* mutants. Each average was recorded from groups of 32 *nf1* mutant larvae. A nonlinear regression was fitted to each distribution. The distribution average and standard deviation was used to calculate *z*-scores to determine compounds that significantly affect behavioral measures. (C) Histograms demonstrating the frequency distribution of *z*-scores calculated for effects of individual compounds. Each average was recorded from groups of 32 treated *nf1* mutant larvae. A *z*-score threshold (2.326) was set representing the one-sided 99% confidence interval to identify compounds that improved habituation or PPI. (D) (Top) Depiction of the modified stimulus assay used to measure habituation. (Bottom) Habituation±s.e.m. in 5 dpf wild-type and *nf1* mutant larvae. Data points represent the average habituation from all larvae tested within each genotype on each experimental day (*n*=8). In total, 148-213 larvae were tested per genotype. One-way ANOVA showed a statistically significant difference between genotypes [*F*(4,35)=23.62, *P*<0.001]. Tukey's adjusted *P*-values were used for comparisons between genotypes. (E) Habituation±s.e.m. for 5 dpf wild-type and *nf1* mutant larvae treated with plerixafor. Data points represent average habituation from all larvae tested within each genotype at each treatment dose (sample sizes: WT control *n*=61, 1 µM *n*=43, 3 µM *n*=53, 10 µM *n*=40; *nf1a^+/−^;nf1b^+/−^* control *n*=38, 1 µM *n*=31, 3 µM *n*=42, 10 µM *n*=19; *nf1a^+/−^;nf1b^−/−^* control *n*=38, 1 µM *n*=34, 3 µM *n*=38, 10 µM *n*=17; *nf1a^−/−^;nf1b^+/−^* control *n*=41, 1 µM *n*=34, 3 µM *n*=37, 10 µM *n*=31; *nf1a^−/−^;nf1b^−/−^* control *n*=50, 1 µM *n*=42, 3 µM *n*=34, 10 µM *n*=29). Two-way ANOVA showed statistically significant differences between treatment groups [*F*(3,732)=31.11, *P*<0.001], as well as an interaction between the genotype and treatment factors [*F*(12,732)=4.017, *P*<0.001]. Dunnet's adjusted *P*-values (below graphs) were used for comparing non-treated larvae within each genotype and comparisons to wild-type larvae within each treatment dose.

In larval zebrafish, acoustic stimuli evoke robust startle responses consisting of ‘C-shape’ bends with distinct kinematic properties (Burgess and Granato, 2007a; Jain et al., 2018; Kimmel et al., 1974). Initiation of a short-latency C-bend (SLC) drives rapid escape behavior (Burgess and Granato, 2007a; Hecker et al., 2020; Jain et al., 2018; Marsden et al., 2018; Wolman et al., 2015). Our behavioral assay was designed to measure baseline startle initiation, PPI and habituation of the zebrafish SLC response in *nf1* mutant larvae. A total of 60 acoustic stimuli were delivered with varying intensities and interstimulus intervals (ISIs) (Fig. 1A). Motor responses of individual larvae at 5 days post fertilization (dpf) were recorded with a high-speed camera and response kinematics were analyzed using automated tracking software. The low-intensity stimulus was designed to evoke SLCs in response to ∼25% of stimuli (Fig. S1A). The high-intensity stimulus was designed to evoke SLCs in response to ∼75% of stimuli (Fig. S1A). PPI was assessed by comparing SLC initiation in response to individual high-intensity stimuli versus high-intensity stimuli that were preceded by low-intensity stimuli (Fig. 1A). Habituation was assessed by comparing SLC initiation in response to high-intensity stimuli separated by 1.5 s ISIs instead of 20 s ISIs (Fig. 1A).

Pharmacological screening of 1134 small-molecule compounds with known biological targets was completed over the course of 60 experimental days. Pair mating *nf1a^+/−^*;*nf1b^−/−^* and *nf1a^−/−^*;*nf1b^+/−^* adults (Shin et al., 2012) yielded a mix of *nf1* mutant embryos with predicted equal ratios of *nf1a^+/−^;nf1b^+/−^*, *nf1a^+/−^;nf1b^−/−^*, *nf1a^−/−^;nf1b^+/−^* and *nf1a^−/−^;nf1b^−/−^* mutant alleles. To determine baseline behavioral responsiveness in non-treated *nf1* mutant larvae, we quantified the baseline initiation of SLC responses and total motor reactions to low-intensity and high-intensity stimuli (Fig. S1A,B). To examine neurofibromin-dependent behaviors in non-treated *nf1* mutant larvae, we quantified habituation to stimuli 41-50, habituation to stimuli 51-60 and PPI (Fig. 1B). Data from two groups of 32 non-treated *nf1* mutant larvae were averaged on each experimental day and used as comparison to identify compounds that significantly regulated acoustically evoked behaviors.

To test the specific behavioral effects of compounds, we first analyzed drugs that altered baseline initiation of total reactions to low or high-intensity stimuli in *nf1* mutant larvae. Forty-nine compounds increased baseline initiation of total reactions to low-intensity stimuli (Fig. S1C; Table S1), and 87 compounds decreased baseline initiation of total turns to high-intensity stimuli (Fig. S1C; Table S1). We then performed enrichment analysis to identify classes of compounds with similar bioactivity by using biological targets defined by the compound library manufacturer (Selleck, https://www.selleckchem.com/screening-libraries.html). Compounds that increased baseline initiation of total reactions to low-intensity stimuli were enriched for retinoid receptor targets (Table 1). Compounds that decreased baseline initiation of total turns to high-intensity stimuli were enriched for estrogen and progestogen receptor, calcium channel and 5-alpha reductase targets (Table 1). Although biologically interesting, we did not pursue compounds that altered baseline initiation of total turns in *nf1* mutant larvae and, instead, focused on compounds that specifically regulated habitation or PPI. Of those, 22 compounds improved habituation to stimuli 41-50 (Fig. 1C; Table S1) and 32 compounds improved habituation to stimuli 51-60 (Fig. 1C; Table S1). Compounds that improved habituation in *nf1* mutant larvae were enriched for adrenergic and 5-hydroxytryptamine (5-HT) receptor targets (Table 1). Additionally, 41 compounds improved PPI (Fig. 1C; Table S1). Compounds that improved PPI were enriched for 5-HT receptor, phosphodiesterase inhibitor and topoisomerase inhibitor targets (Table 1).

**Table 1.** Enrichment analysis for biological targets that regulate acoustically evoked behaviors in *nfl* mutant larvae

Biological targets enriched for increasing total reactions to low-intensity stimuli	
**Enriched biological target**	**Fisher's exact *P*-value**
Retinoid receptor	0.0104

Biological targets enriched for decreasing total reactions to high-intensity stimuli	
**Enriched biological target**	**Fisher's exact *P*-value**
5-alpha reductase	0.0058
Estrogen and progestogen receptor	0.0060
Calcium channel	0.0430

Biological targets enriched for increasing habituation learning	
**Enriched biological target**	**Fisher's exact *P*-value**
Adrenergic (habituation stimuli 51-60)	0.0081
5-HT receptor (habituation stimuli 51-60)	0.0132
5-HT receptor (habituation stimuli 41-50)	0.0245

Biological targets enriched for increasing PPI	
**Enriched biological target**	**Fisher's exact *P*-value**
5-HT receptor	0.0063
Phosphodiesterase inhibitors	0.0166
Topoisomerase inhibitors	0.0393

Lists of biological targets enriched for target compounds that increase initiation of total reactions to low-intensity stimuli, decrease initiation of total reactions to high-intensity stimuli, increase habituation, and increase PPI in *nf1* mutants. Enrichment was determined by Fisher's exact test using a significance level of 0.05. PPI, prepulse inhibition.

Adrenergic and 5-HT signaling pathways are known to regulate habituation learning in wild-type animals (Cassel, 2010; Kocher and Straumann, 2025; Wolman et al., 2011). We were interested in identifying pathways specific to loss of neurofibromin. Therefore, of the drugs that specifically improve habituation and/or PPI without affecting baseline, we focused on plerixafor, a modulator of chemokine-receptor signaling. Plerixafor modulates the CXCL12-CXCR4 and CXCL12-ACKR3 signaling axes through antagonism of CXCR4 or allosteric agonism of ACKR3 (formerly known as CXCR7) (Hatse et al., 2002; Jørgensen et al., 2021; Kalatskaya et al., 2009; Naumann et al., 2010; Sarma et al., 2023; Singh et al., 2013). CXCR4 is an inhibitory G-protein-coupled (G_i_) chemokine receptor and its inhibition has been proposed as a treatment for optic glioma and malignant peripheral nerve sheath tumors (MPNSTs) in NF1 patients (Mo et al., 2013; Warrington et al., 2007). ACKR3 is an atypical chemokine receptor that can scavenge CXCL12 ligand or signal through recruitment of β-arrestin and kinase assembly (Gritsina et al., 2023; Rajagopal et al., 2010; Ray et al., 2012). However, the effects of CXCL12-CXCR4 and CXCL12-ACKR3 signaling on NF1-associated cognitive dysfunction have not been studied. Therefore, we further characterized the effects of plerixafor on habituation in wild-type and *nf1* mutant larvae.

### Cxcr4 signaling regulates habituation in *nfl* mutant larvae

Previous studies of habituation of the SLC response in *nf1* mutant larvae have only identified deficits in *nf1* double homozygous mutants (*nf1a^−/−^;nf1b^−/−^*) (Shin et al., 2012; Wolman et al., 2014). Because the human disease is autosomal dominant and manifests in heterozygous individuals, we established an assay sensitive to habituation deficits in heterozygous *nf1* mutants. We modified our original behavioral assay by removing the PPI phase and increasing the ISI during the pre-habituation (30 s) and the habituation (3 s) phases (Fig. 1D). We tested non-treated wild-type and *nf1* mutant larvae in this modified assay, obtaining an average habituation value from groups of larvae during a total of eight experimental days (Fig. 1D). Larvae were genotyped *post hoc* for *nf1a* and *nf1b* mutant alleles (Shin et al., 2012) and, in total, 148-213 larvae were tested per genotype. One-way ANOVA showed a statistically significant difference between genotypes [*F*(4,35)=23.62, *P*<0.001]. Adjusting for multiple comparisons between genotypes, habituation was significantly higher in wild-type larvae compared to each of the *nf1* mutant groups. Additionally, habituation was significantly lower in *nf1a^−/−^;nf1b^−/−^* mutant larvae compared to each of the three *nf1* heterozygous (*nf1a^+/−^;nf1b^+/−^*, *nf1a^+/−^;nf1b^−/−^*, *nf1a^−/−^;nf1b^+/−^*) mutants (Fig. 1D). Using this modified behavioral assay, we were then able to test if pharmacological modulation of the Cxcl12-Cxcr4 and Cxcl12-Ackr3 signaling axes signaling axis could improve habituation in both *nf1* double homozygous and *nf1* heterozygous mutant larvae.

We tested the effect of Cxcr4 and Ackr3 receptor signaling on habituation by treating larvae with plerixafor and plerixafor 8HCl, two separate formulations of AMD3100 that inhibit Cxcr4 and activate Ackr3 receptor signaling. Using both formulations (Fig. 1E; Fig. S1D), we found significant differences between groups. Plerixafor treatment (Fig. 1E) improved habituation in all *nf1* mutant genotypes without affecting wild-type habituation – restoring habituation to near wild-type levels in *nf1a^+/−^;nf1b^+/−^*, *nf1a^+/−^;nf1b^−/−^* and *nf1a^−/−^;nf1b^−/−^* mutant larvae. Treatment with plerixafor 8HCl (Fig. S1D) significantly improved habituation in *nf1a^−/−^;nf1b^−/−^* and *nf1a^+/−^;nf1b^−/−^* mutant larvae. Since plerixafor is known to modulate both Cxcr4 and Ackr3 receptor signaling with varying effects on downstream signaling (Bonecchi and Graham, 2016; Jørgensen et al., 2021; Lu et al., 2024; Tian et al., 2019), we next sought to differentiate between the two receptor signaling pathways. We tested the effect of Ackr3 activation on habituation by treating larvae with VUF11207, an ACKR3 agonist that induces recruitment of β-arrestin to ACKR3 without altering CXCR4 G-protein activity (Sarma et al., 2023; Wijtmans et al., 2012). We found that VUF11207 did not significantly alter habituation in wild-type or *nf1* mutant larvae (Fig. S2A), supporting the conclusion that plerixafor is acting through Cxcr4 and not Ackr3 signaling to improve habituation. Overall, these pharmacological results support the conclusion that Cxcr4 signaling regulates habituation in *nf1* mutant larvae.

### Inhibition of Cxcr4 does not affect general motor performance at doses that improve habituation

**Fig. 2.**
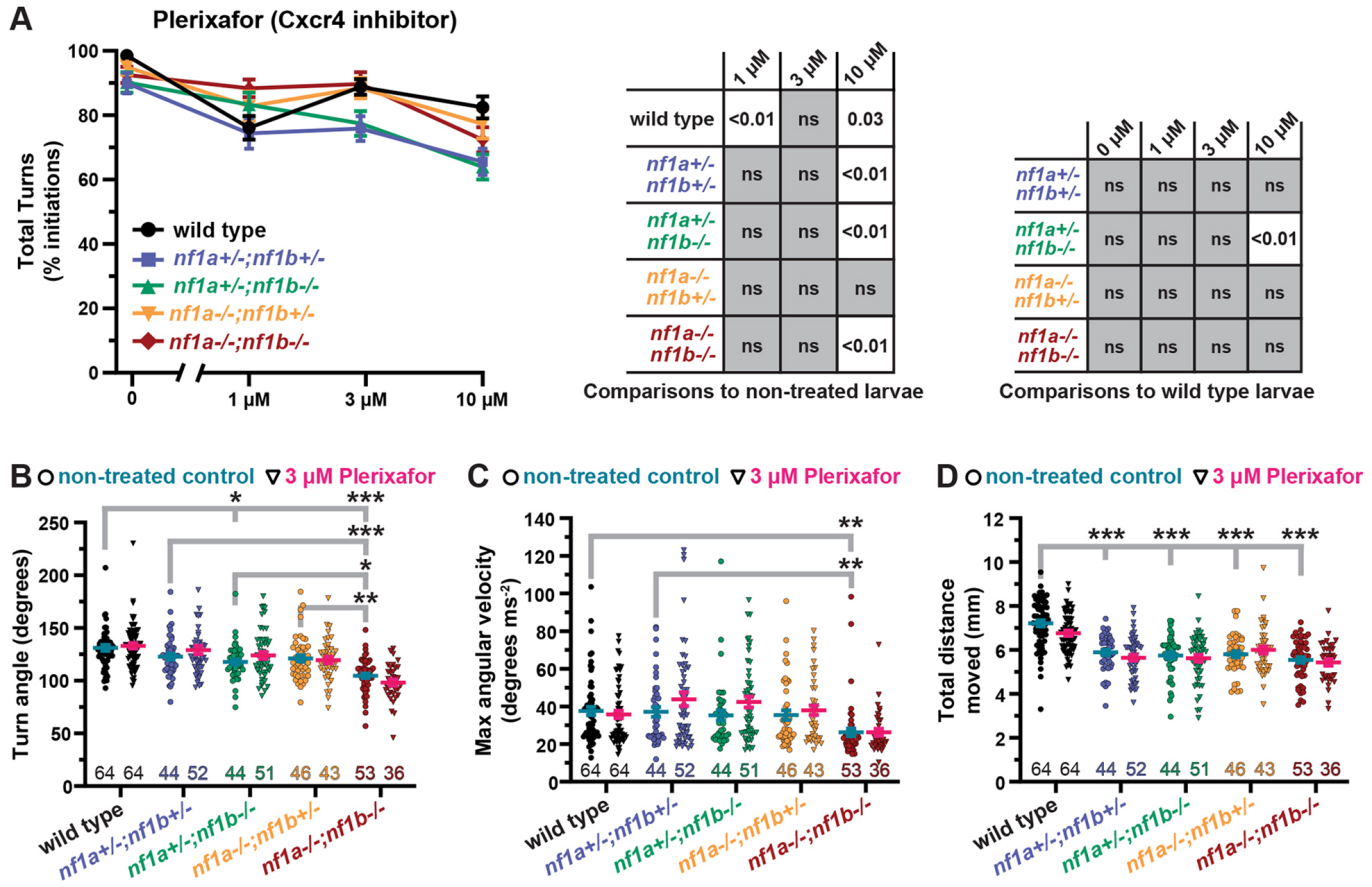
**Inhibition of Cxcr4 does not affect general motor performance at doses that improve habituation.** (A) Percent initiation of total reactions±s.e.m. in 5 dpf wild-type and *nf1* mutant larvae treated with plerixafor. Data points represent average initiation percentage from all larvae tested within each genotype at each treatment dose (sample sizes: WT control *n*=63, 1 µM *n*=64, 3 µM *n*=63, 10 µM *n*=62; *nf1a^+/−^;nf1b^+/−^* control *n*=44, 1 µM *n*=45, 3 µM *n*=53, 10 µM *n*=39; *nf1a^+/−^;nf1b^−/−^* control *n*=44, 1 µM *n*=39, 3 µM *n*=50, 10 µM *n*=52; *nf1a^−/−^;nf1b^+/−^* control *n*=45, 1 µM *n*=44, 3 µM *n*=42, 10 µM *n*=42; *nf1a^−/−^;nf1b^−/−^* control *n*=53, 1 µM *n*=51, 3 µM *n*=37, 10 µM *n*=50). Two-way ANOVA showed statistically significant differences between treatment groups [*F*(3,414)=11.27, *P*<0.001], as well as a statistically significant interaction between genotype and treatment factors [*F*(12,962)=1.907, *P*=0.030]. Dunnet's adjusted *P*-values were used for comparisons between non-treated larvae within each genotype. (B-D) Turn angle, maximum angular velocity and total distance moved in response to baseline high-intensity stimuli±s.e.m. in 5 dpf wild-type and *nf1* mutant larvae treated with 3 µM plerixafor. Data points represent average values from individual larvae in response to ten high-intensity stimuli. Sample sizes are indicated with color-coded numbers along the *x*-axis. Tukey's adjusted *P*-values were used for comparing genotypes. (B) Two-way ANOVA showed statistically significant differences in turn angle between genotypes [*F*(4,487)=27.80, *P*<0.001] but not plerixafor treatment [*F*(1,487)=0.4121, *P*=0.521]. (C) Two-way ANOVA showed statistically significant differences in maximum angular velocity between genotypes [*F*(4,487)=8.309, *P*<0.001] but not plerixafor treatment [*F*(1,487)=3.100, *P*=0.079]. (D) Two-way ANOVA showed statistically significant differences in total distance moved between genotypes [*F*(4,487)=42.29, *P*<0.001] but not plerixafor treatment [*F*(1,487)=2.735, *P*=0.099]. *P<0.05, **P<0.01, ***P<0.001.

To test the behavioral specificity of Cxcr4 inhibition at different treatment doses, we analyzed the effect of plerixafor on general motor performance by quantifying initiation of total turns to pre-habituation high-intensity stimuli. At the 3 µM dose for plerixafor and 300 nM dose for plerixafor 8HCl, treatment did not significantly affect baseline initiation of total turns to high-intensity stimuli in wild-type larvae (Fig. 2A; Fig. S3A). Therefore, we selected the 3 µM dose of plerixafor as a treatment that selectivity improves habituation in *nf1* mutant larvae (Fig. 1E) without affecting baseline initiation of turns (Fig. 2A). All subsequent experiments were conducted comparing non-treated and 3 µM plerixafor-treated larvae.

In a previous report on *nf1* mutant larvae, *nf1a^−/−^;nf1b^−/−^* mutants displayed motor deficits in addition to their habituation learning deficits, with kinematically weaker SLC responses as measured by decreased head turn angle, maximum angular velocity, and total distance traveled (Shin et al., 2012). To test if Cxcr4 inhibition affects the kinematic properties of turning behavior, we analyzed turn kinematics in non-treated and 3 µM plerixafor-treated larvae (Fig. 2B-D). Consistent with the previous report (Shin et al., 2012), we observed kinematically weaker SLC responses for turn angle, maximum angular velocity and total distance moved in *nf1a^−/−^;nf1b^−/−^* larvae. Additionally, we found a decrease in total distance moved for each of the *nf1* heterozygous mutant larvae and a decrease in turn angle in *nf1a^+/−^;nf1b^−/−^* larvae. However, these motor deficits were not improved by inhibiting Cxcr4. Therefore, these results support a conclusion that Cxcr4 inhibition specifically regulates habituation learning in *nf1* mutants without altering general motor performance.

### Gene expression analysis reveals *cxcr4b* is upregulated in *nf1* double homozygous mutants

We used a second approach, RNAseq analysis, to identify additional or overlapping molecular targets influenced by loss of neurofibromin. RNA was isolated from whole larvae in wild-type and putative *nf1* double homozygous mutant larvae identified by hyperpigmentation phenotype (see Materials and Methods). Differential gene expression analysis followed by KEGG pathway enrichment analysis identified multiple metabolic pathways enriched for genes upregulated in *nf1* double homozygous mutants (Table 2). Focusing on Cxcr4 signaling, differential expression revealed upregulation of *cxcr4b* at 5 dpf in *nf1* double homozygous mutants compared to wild-type larvae (fold change=1.52, adjusted *P*-value<0.001). The increased expression of *cxcr4b* in *nf1* double homozygous mutant larvae alongside the small molecule drug screen results lead us to investigate the relationship between Cxcr4 and neurofibromin signaling.

**Table 2.** KEGG pathway analysis for differentially expressed genes in *nf1* double homozygous mutants

KEGG pathway	Adjusted *P*-value	Gene count
Retinol metabolism	1.80E-11	18
Drug metabolism – cytochrome P450	4.87E-10	15
Metabolism of xenobiotics by cytochrome P450	9.09E-10	15
Pentose and glucuronate interconversions	1.16E-09	13
Ascorbate and aldarate metabolism	1.52E-09	13
Porphyrin and chlorophyll metabolism	7.09E-09	13
Steroid hormone biosynthesis	7.09E-09	14
PPAR signaling pathway	6.70E-08	14
Drug metabolism – other enzymes	2.59E-07	14
Carbon metabolism	4.48E-05	13
Glycolysis/Gluconeogenesis	0.000361	9
Fatty acid degradation	0.001064	7
Glycine, serine and threonine metabolism	0.003035	6
Glyoxylate and dicarboxylate metabolism	0.006859	5
Alpha-linolenic acid metabolism	0.006904	4
Linoleic acid metabolism	0.010676	4
Biosynthesis of amino acids	0.016604	7
Pyruvate metabolism	0.016604	5
ABC transporters	0.016604	5
Alanine, aspartate and glutamate metabolism	0.017284	5
Glycerolipid metabolism	0.018052	6
Tyrosine metabolism	0.040536	4
Pantothenate and CoA biosynthesis	0.047723	3

Lists of Kyoto Encyclopedia of Genes and Genomes (KEGG) pathways with overrepresentation of differentially expressed genes from RNAseq analysis comparing 5-dfp *nf1* double homozygous mutants to wild-type larvae.

### Cxcr4 signaling has limited effects on canonical Ras-Raf-MEK-ERK signaling in the brain

CXCR4 is a G_i_ protein-coupled chemokine receptor specific for the ligand stromal-derived-factor-1 (CXCL12, also known as SDF1). Through Gα_i_ protein activation, the CXCR4 receptor is known to increase Ras signaling and inhibit cAMP signaling (Busillo and Benovic, 2007). Neurofibromin is also known to regulate these signaling pathways but in the opposing directions – by inhibiting Ras signaling and increasing cAMP signaling. Therefore, in neurofibromin-deficient larvae, we predicted Cxcr4 inhibition might decrease overactive Ras signaling and/or increase suppressed cAMP signaling.

**Fig. 3.**
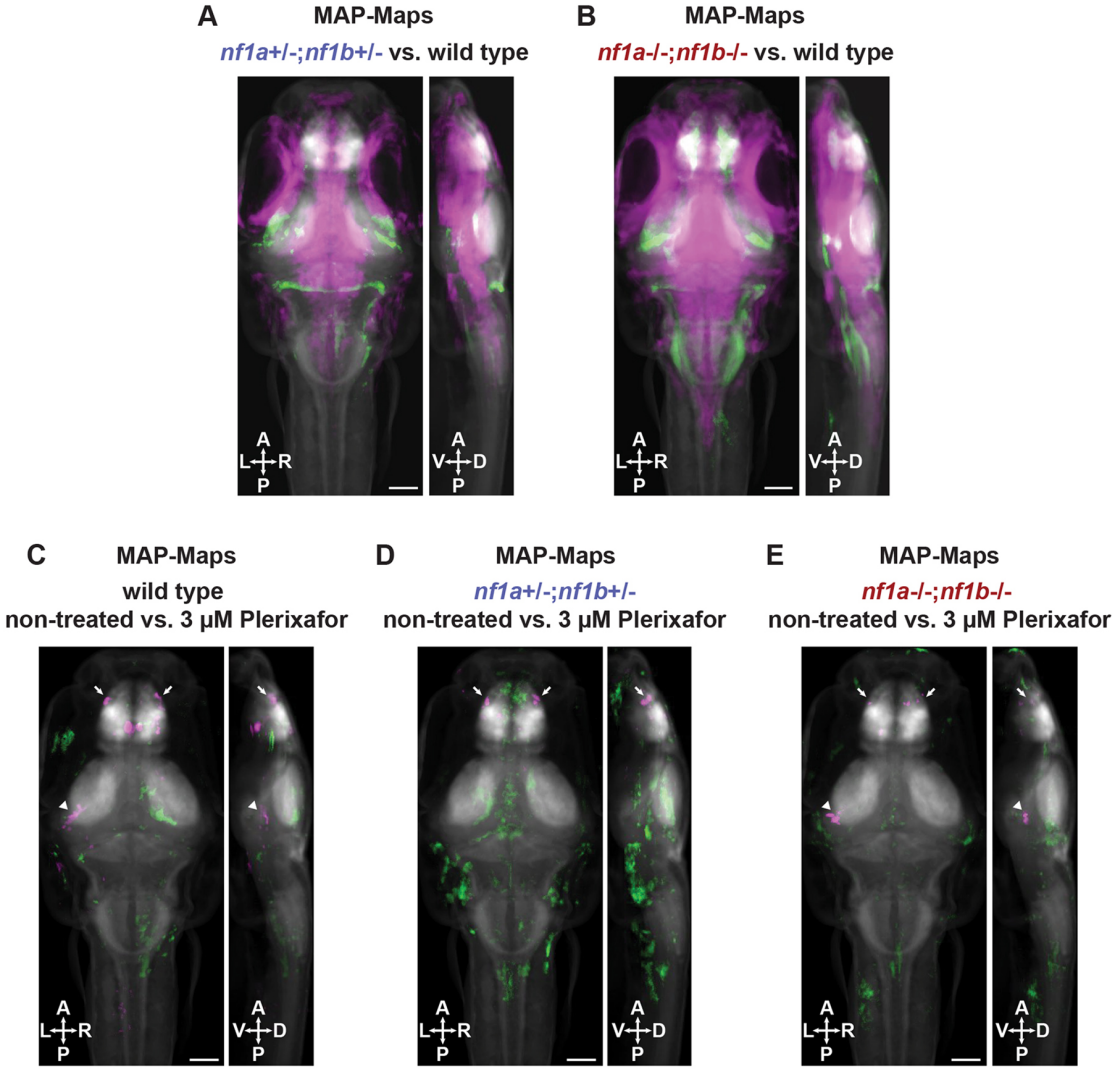
**MAP-mapping of pERK shows that Cxcr4 inhibition has limited effect on canonical Ras signaling in the brain.** (A,B) MAP-maps showing statistically significant median *z*-score differences in non-treated (A) *nf1a^+/−^;nf1b^+/−^* versus wild-type larvae and (B) *nf1a^−/−^;nf1b^−/−^* versus wild-type larvae. Green signals represent increased pERK levels in *nf1* mutant larvae compared to wild type. Magenta signals denote decreased levels of phosphorylated ERK1/2 (pERK) in *nf1* mutant larvae compared to those in wild type. (C,E) Genotype-matched plerixafor treatment. MAP-maps showing statistically significant median *z*-score differences in non-treated versus 3 µM plerixafor-treated wild-type (C), *nf1a^+/−^;nf1b^+/−^* (D) and *nf1a^−/−^;nf1b^−/−^* (E) larvae. Green signals represent increased pERK levels in plerixafor-treated compared to non-treated larvae within each genotype. Magenta signals represent decreased pERK levels in plerixafor-treated compared to non-treated larvae within each genotype. Arrowheads indicate the torus semicircularis region of interest (ROI) within the midbrain. Arrows indicate the olfactory bulb ROI. All larvae were fixed and stained at 6 dpf. The *z*-scores were calculated using Mann–Whitney *U* statistics with the significance threshold set by using a false discovery rate (FDR) of 0.00005. *n*=18-20 larvae per treatment group. A, anterior; D, dorsal; P, posterior; V, ventral. Scale bars: 200 µM.

We first tested where overactive Ras signaling is present in the brains of *nf1* mutant larvae. The canonical Ras signaling pathway involves a kinase cascade that phosphorylates the mitogen-activated protein kinase (MAPK) family members MAPK1 and MAPK3 [also known as extracellular signal-regulated kinases 2 and 1 (ERK2 and ERK1), respectively] (Lavoie et al., 2020). Phosphorylation of ERK1/2 can be detected and quantified in larval zebrafish brains using immunolabeling and the open-source Z-BRAIN reference atlas (Randlett et al., 2015). This technique, named MAP-mapping, is often used to detect changes in neuronal activity as calcium influx activates ERK1/2 signaling (Randlett et al., 2015; Rosen et al., 1994; Thomas and Huganir, 2004). Here, differences in phosphorylation of ERK1/2 between experimental groups may result from direct loss of neurofibromin altering Ras-Raf-MEK-ERK signaling or the indirect effects of altered neuronal activity in *nf1* mutants. We fixed freely swimming larvae at 6 dpf, immunostained for total ERK1/2 (tERK) and phosphorylated ERK1/2 (pERK), and then quantified and mapped images from wild-type, *nf1a^+/−^;nf1b^+/−^* and *nf1a^−/−^;nf1b^−/−^* larvae. Comparing *nf1a^+/−^;nf1b^+/−^* larvae to wild type (Fig. 3A) and *nf1a^−/−^;nf1b^−/−^* larvae to wild type (Fig. 3B), we found region-specific increases and decreases in levels of pERK. We focused on regions of interest associated with acoustic startle that displayed increased amounts of pERK (Table 3), an indicator that loss of neurofibromin may be contributing to overactivation of Ras in these regions. Acoustic startle responses in zebrafish are triggered by activation of reticulospinal Mauthner neurons in the hindbrain, which integrate acoustic and vibrational inputs from the statoacoustic ganglia and the lateral line and send output signals to motor neurons to induce muscle contraction (Eaton et al., 1977; Hecker et al., 2020; Liu and Fetcho, 1999; Wolman et al., 2015). Additionally, the octavolateralis nuclei and torus semicircularis relay auditory information to the thalamus. Some auditory information from the torus semicircularis and thalamus is also sent back to the hindbrain in zebrafish (Vanwalleghem et al., 2017) and could, potentially, provide top-down regulation of hindbrain activity to regulate acoustic startle habituation. Compared to wild type, *nf1a^+/−^;nf1b^+/−^* and *nf1a^−/−^;nf1b^−/−^* larvae showed increased amounts of pERK in the torus semicircularis and hindbrain, indicating that Ras signaling may be dysregulated in these regions. Therefore, we next asked if Cxcr4 inhibition decreases pERK in these brain regions.

**Table 3.** MAP-mapping ROI analyses for *nf1* mutant versus wild-type larvae

ROI analysis: increased pERK/tERK in *nf1a+/−;nf1b+/−* mutants vs wild type							
ROI	Signal in ROI	Cell-type label 1	Signal 1	Cell-type label 2	Signal 2	Cell-type label 3	Signal 3
Torus semicircularis	426.6499	anti-5-HT	2.4756	anti-Gad67	2.1449	anti-GlyR	2.1384
Telencephalon (anterior forebrain)	2.1172	anti-tERK	4.6125	anti-5-HT	4.1013	EtVmat2-GFP	3.5844
Diencephalon (posterior forebrain)	35.4359	Vglut2a-GFP	2.4285	anti-tERK	1.9462	anti-Zrf2	1.5595
Mesencephalon (midbrain)	193.4313	Isl2bGal4-uasDendra	30.6283	anti-Znp1	9.9624	anti-Zrf2	6.922
Rhombencephalon (hindbrain)	131.834	Ptf1aGal4-uasKaede	7.188	anti-Znp1	5.7143	anti-Zn1	5.6403

ROI analysis: increased pERK/tERK in *nf1a−/−;nf1b−/−* mutants vs wild type							
ROI	Signal in ROI	Cell-type label 1	Signal 1	Cell-type label 2	Signal 2	Cell-type label 3	Signal 3
Torus semicircularis	1290.011	anti-GlyR	2.7734	anti-5-HT	2.7449	anti-Gad67	2.4702
Telencephalon (anterior forebrain)	1307.928	anti-Zrf2	6.5138	anti-tERK	5.8422	anti-Zn1	4.4325
Diencephalon (posterior forebrain)	501.0858	anti-Zrf2	2.619	anti-tERK	2.4348	Vglut2a-GFP	2.0668
Mesencephalon (midbrain)	344.4349	Isl2bGal4-uasDendra	15.7938	anti-Znp1	6.6898	anti-Zrf2	5.4395
Rhombencephalon (hindbrain)	635.2197	anti-GlyR	9.0154	anti-Znp1	5.0124	anti-Zrf2	4.4444

Quantification of the mean *z*-score difference between genotype groups in each region of interest (ROI) is reported as Signal in ROI. The top three Z-BRAIN labels that show overlap with the signal for each ROI are reported. Reported signal values of >1 indicate relative enrichment.

To investigate whether Cxcr4 inhibition modulates Ras signaling in brain regions associated with acoustic startle, we performed MAP-mapping on wild-type (Fig. 3C), *nf1a^+/−^;nf1b^+/−^* (Fig. 3D) and *nf1a^−/−^;nf1b^−/−^* (Fig. 3E) larvae comparing non-treated to plerixafor-treated groups within each genotype (Table 4). Overall, plerixafor treatment had limited effects on pERK levels in both wild-type and *nf1* mutant larvae. We did find decreased levels of pERK in the torus semicircularis of plerixafor-treated wild-type and *nf1a^−/−^;nf1b^−/−^* larvae compared to non-treated larvae. However, these differences were unilateral and not observed in *nf1a^+/−^;nf1b^+/−^* larvae (Fig. 3D). Additionally, plerixafor treatment had very limited effects on pERK in the hindbrain (Table 4), where Mauthner neuron circuitry drives acoustic startle responses. Interestingly, the olfactory bulb showed decreased levels of pERK in both wild-type and *nf1* mutant larvae treated with plerixafor (Fig. 3C-E; Table 4). This is consistent with studies of larval zebrafish and adult mice showing *Cxcr4* mRNA is highly expressed in the olfactory bulb (Förster et al., 2017; Stumm et al., 2002). However, this result is unlikely to be related to regulation of acoustic startle responses. Taken together, these results support the conclusion that Cxcr4 signaling has a limited role in modulating canonical Ras-Raf-MEK-ERK signaling in the larval zebrafish brain.

**Table 4.** MAP-mapping ROI analyses of plerixafor-treated versus non-treated larvae

ROI analysis: decreased pERK/tERK in wild-type plerixafor-treated vs non-treated							
ROI	Signal in ROI	Cell-type label 1	Signal 1	Cell-type label 2	Signal 2	Cell-type label 3	Signal 3
Torus semicircularis	97.6712	anti-5-HT	3.2096	anti-GlyR	2.9163	anti-Gad67	2.5256
Olfactory bulb	206.6698	Vglut2a-GFP	7.5826	Elavl3-GCaMP5G	4.3585	Elavl3-H2BRFP	4.1155
Telencephalon (anterior forebrain)	91.6901	anti-5-HT	5.874	anti-tERK	5.866	anti-Zrf2	4.9997
Diencephalon (posterior forebrain)	49.4953	anti-Zrf2	1.9229	Gad1b-GFP	1.8527	anti-5-HT	1.4151
Mesencephalon (midbrain)	18.2628	anti-Znp1	3.5465	anti-GlyR	3.5321	anti-5-HT	2.1924
Rhombencephalon (hindbrain)	2.0154	anti-GlyR	2.775	Elavl3-H2BRFP	2.3555	Vglut2a-GFP	1.7154

ROI analysis: decreased pERK/tERK in *nf1a+/−;nf1b+/−* plerixafor-treated vs non-treated							
ROI	Signal in ROI	Cell-type label 1	Signal 1	Cell-type label 2	Signal 2	Cell-type label 3	Signal 3
Olfactory bulb	425.4882	Vglut2a-GFP	7.4568	Elavl3-GCaMP5G	4.0149	anti-Gad67	3.0747
Telencephalon (anterior forebrain)	63.0717	Vglut2a-GFP	5.8461	anti-tERK	5.4957	anti-5-HT	5.39
Diencephalon (posterior forebrain)	2.0858	Vglut2a-GFP	4.3825	anti-tERK	2.0935	anti-5-HT	1.8519
Rhombencephalon (hindbrain)	0.17789	anti-Zrf1(GFAP)	3.1122	anti-GlyR	2.298	anti-5-HT	1.57

ROI analysis: decreased pERK/tERK in *nf1a−/−;nf1b−/−* plerixafor-treated vs non-treated							
ROI	Signal in ROI	Cell-type label 1	Signal 1	Cell-type label 2	Signal 2	Cell-type label 3	Signal 3
Torus semicircularis	87.7413	anti-5-HT	3.1665	anti-GlyR	3.038	anti-Gad67	2.9835
Olfactory Bulb	28.3954	Vglut2a-GFP	6.9219	anti-5-HT	2.6627	anti-Gad67	2.4144
Telencephalon (anterior forebrain)	10.7501	Elavl3-H2BRFP	4.1704	anti-5-HT	4.0196	Vglut2a-GFP	3.5166
Diencephalon (posterior forebrain)	1.0461	anti-Zrf2	4.6717	anti-tERK	4.0909	anti-5-HT	2.7117
Mesencephalon (midbrain)	11.6061	anti-Znp1	4.7141	anti-GlyR	4.2096	anti-Gad67	2.3958

Quantification of the mean *z*-score difference between treatment groups in each region of interest (ROI) is reported as ‘Signal in ROI’. The top three Z-BRAIN labels that show overlap with the signal for each ROI are reported. Reported signal values of >1 indicate relative enrichment.

### Cxcr4 signaling increases downregulated cAMP-PKA signaling in *nf1* heterozygous mutants

**Fig. 4.**
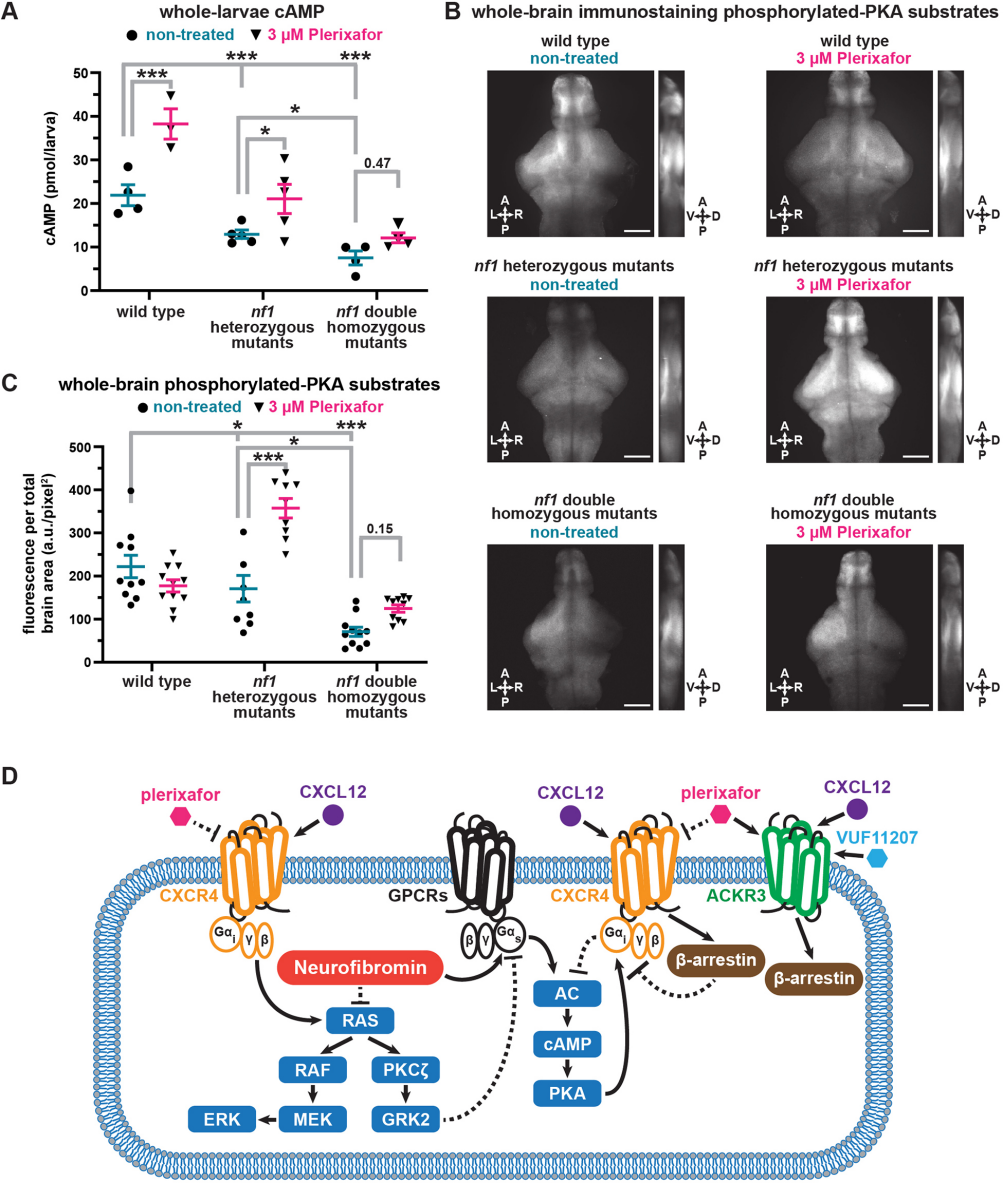
**cAMP ELISA and phosphorylated PKA substrate immunofluorescence show Cxcr4 inhibition increases downregulated cAMP-PKA signaling in *nf1* mutants.** (A) cAMP levels±s.e.m. in 5 dpf wild-type and *nf1* mutant larvae treated with 3 µM plerixafor. Data points represent averages of 3 technical replicates (sample sizes: WT control *n*=4, treated *n*=3; *nf1* heterozygous mutant control *n*=5, treated *n*=5; *nf1* double homozygous mutant control *n*=4, treated *n*=4). Two-way ANOVA showed statistically significant differences between genotypes [*F*(2,19)=33.79, *P*<0.001] and treatment groups [*F*(1,19)=24.87, *P*<0.001]. Tukey's and Šídák's adjusted *P*-values were used for multiple comparisons. (B) Representative phosphorylated-PKA substrate immunofluorescent images. Average intensity summation projections covering the whole brain. A, anterior; D, dorsal; P, posterior; V, ventral. Scale bars: 100 µM. (C) Phosphorylated-PKA substrate immunofluorescence. Values reported as arbitrary units of immunofluorescence per total brain area after subtracting background. Data points represent measurements from individual larvae (sample sizes: WT control *n*=10, treated *n*=11; *nf1* heterozygous mutant control *n*=8, treated *n*=9; *nf1* double homozygous mutant control *n*=11, treated *n*=10). Two-way ANOVA showed statistically significant differences between genotypes [*F*(2,54)=35.72, *P*<0.001], treatments [*F*(1,54)=16.41, *P*<0.001], and a statistically significant interaction between genotype and treatment [*F*(2,54)=16.83, *P*<0.001]. Tukey's adjusted *P*-values were used for multiple comparisons. *P<0.05, ***P<0.001. (D) Cellular signaling schematic highlighting interactions between drug treatments and known neurofibromin, CXCR4, and ACKR3 signaling mechanisms. Loss of neurofibromin is predicted to increase RAS and decrease cAMP signaling. Conversely, plerixafor treatment inhibits CXCR4 receptor signaling by blocking CXCL12 binding and stimulating β-arrestin recruitment which is predicted to decrease RAS and increase cAMP signaling. Our results show that in larval zebrafish, *nf1* mutants have decreased cAMP-PKA signaling and that plerixafor treatment increases cAMP and PKA signaling in *nf1* heterozygous mutants. A known negative feedback mechanism in which PKA undergoes cAMP-dependent stimulation of Gαi signaling could explain the interaction between plerixafor treatment, neurofibromin, and CXCR4 signaling. In cases where AC signaling is very low, like in *nf1* double homozygous mutants, Gαi inhibition may also be decreased due to diminished cAMP-PKA-dependent negative feedback. Therefore, plerixafor treatment, which is predicted to block Gαi inhibition of AC would have limited effect. Alternatively, in the case of *nf1* heterozygous mutants, where cAMP-PKA signaling is reduced to a lesser extent, there may still exist meaningful cAMP-PKA-dependent negative feedback. Therefore, blocking Gαi inhibition of AC with plerixafor has a measurable effect.

Previous studies indicate that neurofibromin-deficient mice, fruit flies and zebrafish have reduced cAMP levels (Brown et al., 2010b; Ho et al., 2007; Tong et al., 2002; Wolman et al., 2014). Consistent with these results, we found decreased cAMP levels in *nf1* mutants compared to wild-type larvae when using enzyme-linked immunosorbent assay (ELISA) to quantify cAMP (Fig. 4A). We collected tissue for ELISA analyses from 5 dpf wild-type and *nf1* mutant larvae separated into *nf1* heterozygous mutant and *nf1* double homozygous mutant groups by hyperpigmentation phenotype. We found that cAMP levels were significantly lower in *nf1* heterozygous mutants compared to wild-type larvae and lower in *nf1* double homozygous mutants compared to both *nf1* heterozygous mutants and wild-type larvae (Fig. 4A). Next, we asked if Cxcr4 inhibition increased cAMP levels in *nf1* mutant larvae. We found that plerixafor treatment increased cAMP levels in wild-type and *nf1* heterozygous mutant larvae but not in *nf1* double homozygous mutant larvae (Fig. 4A). This finding suggests that CXCR4 inhibition of cAMP has a neurofibromin-dependent component, as plerixafor does not significantly increase cAMP levels in the complete absence of neurofibromin. Taken together, these results support the conclusion that all *nf1* mutants have suppressed cAMP levels but that Cxcr4 inhibition raises cAMP levels in *nf1* heterozygous mutant larvae.

To regulate habituation of the SLC response, Cxcr4 signaling would need to modulate cAMP in neural circuits associated with acoustic startle. Therefore, we asked if Cxcr4 inhibition increases cAMP signaling in the brain. The cAMP effector protein kinase A (PKA) phosphorylates protein targets to regulate diverse cellular processes (Taskén and Aandahl, 2004). Using an antibody that recognizes substrates phosphorylated by PKA (pPKA-substrates) (Schmitt and Stork, 2002), we performed whole-brain immunolabeling on 5 dpf wild-type and *nf1* mutant larval brains. *nf1* heterozygous mutant and *nf1* double homozygous mutant larvae were separated into groups by hyperpigmentation phenotype. To enable the pPKA-substrate antibody to penetrate the brain, we dissected brain tissue from larvae and used a staining protocol that prevented us from registering images to the Z-BRAIN reference atlas. Instead, we created average fluorescence summation projections (Fig. 4B) to quantify pPKA-substrate fluorescence in whole brains (Fig. 4C). We found that *nf1* heterozygous mutants have significantly lower pPKA-substrate fluorescence than wild-type larvae and that *nf1* double homozygous mutants have significantly lower pPKA-substrate fluorescence than wild-type and *nf1* heterozygous mutants. With plerixafor treatment, we found significantly increased pPKA-substrate fluorescence in *nf1* heterozygous mutant larvae only. These results are consistent with the whole-body cAMP measurements in that all *nf1* mutants have suppressed pPKA levels and Cxcr4 inhibition raises pPKA levels in *nf1* heterozygous mutant larvae. Overall, this supports the conclusion that downregulated cAMP-PKA signaling in neurofibromin-deficiency can be rescued by Cxcr4 inhibition in *nf1* heterozygous mutants, which model the autosomal dominant human disease.

## DISCUSSION

Translation of research in animal models into treatments for NF1-associated cognitive dysfunction has not yet been successful, in part due to the complicated, multi-functional roles that neurofibromin plays in the distinct neural circuits and intracellular signaling cascades that regulate cognitive function. Here, we identify Cxcr4 chemokine receptor signaling as a regulator of habituation, a sensory filtering behavior and attention-based learning process similar to the cognitive behavior processes that are impaired in NF1 patients. While Cxcr4 signaling can regulate both the Ras and cAMP signaling pathways, we find that Cxcr4 predominantly affects cAMP-PKA signaling in *nf1* mutant larval zebrafish. Previous work showed that CXCR4 expression is elevated in malignant peripheral nerve sheath tumors (MPNSTs), and CXCR4 signaling was suggested as a target for treating neurofibromin-deficient tumorigenesis (Mo et al., 2013; Warrington et al., 2007). However, roles in NF1 cognitive dysfunction have not been explored. Our results provide evidence that Cxcr4 signaling may also regulate neurofibromin-dependent cognitive function and, thus, is a potential therapeutic target for NF1 cognitive deficits.

To identify molecular pathways that regulate neurofibromin-dependent behaviors, we chose to measure PPI and habituation of the SLC response in larval zebrafish. PPI and habituation learning deficits have previously been identified in neurofibromin-deficient mice and homozygous mutant zebrafish (Li et al., 2005; Randlett et al., 2019; Shin et al., 2012; Wolman et al., 2014). To develop a model that more closely resembles the heterozygous loss-of-function mutations observed in patients with NF1, we modified our behavioral assay to increase the interstimulus interval between acoustic stimuli during the habituation phase. This modification revealed behavioral deficits in *nf1* heterozygous mutants with at least one functioning allele of *nf1*, thus allowing us to better model the human disease.

Our behavioral drug screening experiments implicate Cxcr4 signaling as a modifier of behavioral deficits in *nf1* mutants. By testing multiple doses of two formulations of the Cxcr4 inhibitor plerixafor in *nf1* mutants, we found robust increases in habituation at doses that did not affect baseline reactions to high-intensity stimuli, and that did not alter the kinematic properties of turning behavior. These findings suggest that Cxcr4 signaling specifically regulates habituation in *nf1* mutants without altering general motor performance. An important caveat to pharmacological approaches is the possibility for off-target effects, and indeed plerixafor, first thought to be a specific Cxcr4 inhibitor, can also stimulate Ackr3 (formerly Cxcr7) at higher concentrations. Our data showing that the specific Ackr3 agonist VUF11207 did not rescue habituation in *nf1* mutants supports the idea that the action of plerixafor in *nf1* mutants is via Cxcr4 inhibition.

NF1 and Cxcr4 signaling both affect the Ras-Raf-MEK-ERK and cAMP-PKA signaling pathways, but in opposite directions; NF1 decreases Ras activity and increases cAMP activity, while Cxcr4 increases Ras activity and suppresses cAMP activity. Thus, the finding that Cxcr4 inhibition rescues *nf1* mutant behavioral defects is, perhaps, not surprising but prompts the question of the molecular mechanisms by which Cxcr4 signaling influences behavior in *nf1* mutants. Our finding that cAMP and PKA signaling were reduced in *nf1* mutant larvae, while increases in phosphorylation of ERK1/2 were limited and region-specific, suggests that cAMP signaling is the primary pathway influencing habituation behavior. These results are consistent with past work that investigated the molecular basis for learning and memory deficits in *nf1* mutant zebrafish and *Drosophila*, and found that learning defects were rescued by manipulations that increase cAMP levels, while memory defects were rescued by inhibition of Ras signaling (Brown et al., 2010b; Ho et al., 2007; Tong et al., 2002; Wolman et al., 2014).

Our finding that Cxcr4 inhibition increased whole-body cAMP levels and brain pPKA-substrate immunofluorescence in *nf1* heterozygous but not homozygous larvae suggests that a component of the Cxcr4 effect on cAMP and pPKA is neurofibromin-dependent. One potential explanation for this result is a negative feedback mechanism in which cAMP-bound PKA regulatory subunits interact with Gα_i_ and enhance Gα_i_ signaling (Stefan et al., 2011) (Fig. 4D). High cAMP levels inhibit further adenylyl cyclase activity by potentiating G_i_ protein-coupled receptors, such as Cxcr4. Therefore, the greater effect of Cxcr4 inhibition on cAMP-PKA signaling in *nf1* heterozygous compared to *nf1* homozygous larvae may be due to the higher baseline cAMP levels in heterozygous animals generated by the presence of some functioning neurofibromin. Surprisingly, we found that plerixafor treatment in wild-type larvae did not have a measurable effect on pPKA immunofluorescence in the brain. This finding may reflect a key role for neurofibromin in maintaining signaling pathway homeostasis. Under wild-type conditions and normal neurofibromin levels, the effects of plerixafor treatment on pPKA substrates may be transient and not detectable with the whole-brain immunohistochemistry method. Cxcr4 and neurofibromin both influence multiple downstream signal transduction pathways with extensive possibilities for cross-talk and feedback within pathways. This complexity underscores the need for continued research into molecular mechanisms of NF1 disease.

It is interesting that total loss of neurofibromin in *nf1* double homozygous mutants does not lead to more global increases of pERK levels, as *nf1a* and *nf1b* are broadly expressed in the larval zebrafish brain (Padmanabhan et al., 2009). One possible explanation is that phosphorylation of ERK1/2 could be regulated by altered neuronal calcium signaling in addition to neurofibromin-dependent increases in Ras signaling *nf1* mutants (Rosen et al., 1994; Thomas and Huganir, 2004). Dysregulation of intracellular calcium in neurons could potentially diminish the hyperactivation of Ras caused by loss of neurofibromin. Previous work in mice shows that loss of neurofibromin can lead to increased gamma aminobutyric acid (GABA) release in neural circuits (Cui et al., 2008; Shilyansky et al., 2010), which could inhibit calcium influx and thus inhibit the activation of Ras in affected brain regions.

Through MAP-mapping analysis, we did identify one brain region with increased levels of pERK that could, potentially, regulate acoustically evoked startle habituation – the torus semicircularis (Fig. 3A; Table 3). In larval zebrafish, the torus semicircularis relays auditory information to the thalamus but also signals back to the hindbrain (Vanwalleghem et al., 2017), where it could, potentially, provide top-down regulation of hindbrain activity to regulate acoustic startle habituation. However, pERK in this region is not reduced in *nf1* heterozygous mutants by Cxcr4 inhibition (Fig. 3D; Table 4). Therefore, Cxcr4 signaling is unlikely to regulate habituation through modulation of the canonical Ras-Raf-MEK-ERK signaling pathway. This is consistent with a previous report that showed MEK inhibition with U0126 does not improve short-term habituation of the SLC response in larval zebrafish (Wolman et al., 2014). However, neurofibromin has also been shown to regulate cellular signaling through non-canonical Ras pathways (Anastasaki and Gutmann, 2014). Ras can activate atypical protein kinase C zeta (PKCζ) to regulate cAMP homeostasis. If neurofibromin regulates cAMP through a Ras-PKCζ pathway in zebrafish larvae, it would not be detected with the pERK MAP-mapping technique used in this study. Therefore, we cannot rule out a potential role for non-canonical Ras signaling in the dysregulation of cAMP homeostasis we observed in *nf1* mutants.

### Other molecular targets that regulate neurofibromin-dependent behaviors

It is important to consider if the biological actions of the target compounds identified by us are specific to loss of neurofibromin or if they affect behavior more generally. A small-molecule screen measuring short-term habituation of the SLC response has previously been performed in wild-type larvae (Wolman et al., 2011). Target biological pathways identified in both our *nf1* mutant screen and the previous wild-type screen, including the 5-HT receptor pathway, may not be specific to loss of neurofibromin (Table 1; Wolman et al., 2011). Therefore, the effects of modulating serotonin signaling on habituation learning may not be specific to loss of neurofibromin. However, neurofibromin has been shown to modulate 5-HT_6_ receptor constitutive activity in mouse neurons (Deraredj Nadim et al., 2016). Therefore, the relationship between neurofibromin and 5-HT receptor activity warrants further consideration. With respect to the compounds that target adrenergic receptors identified in our screen, the alpha-2 adrenergic receptor agonists are known to have sedative effects in humans and zebrafish (Giovannitti et al., 2015; Ruuskanen et al., 2005). Therefore, the effects of these compounds also may not be specific to habituation.

The compounds identified in our screen that improved PPI in *nf1* mutants were enriched for those targeting biological pathways that include 5-HT receptors, phosphodiesterase inhibitors and topoisomerase inhibitors (Table 1). Modulation of 5-HT receptor signaling has previously been shown to regulate PPI in wild-type animals (Fletcher et al., 2001; Rigdon and Weatherspoon, 1992; Sipes and Geyer, 1994). Therefore, the effects of modulating serotonin signaling on PPI may not be specific to loss of neurofibromin. Phosphodiesterase (PDE) inhibitors increase cAMP signaling by preventing the degradation of cAMP. Interestingly, PDE inhibitors have previously been shown to improve habituation in *nf1* double homozygous mutants (Wolman et al., 2014) but their effects on PPI were unknown. Although compounds that improved habituation in our screen were not enriched for PDE inhibitors, we did identify rolipram and doxofylline as two PDE inhibitors that improve habituation in *nf1* mutants (Table S1). Topoisomerases bind to and cut the DNA phosphate backbone and are used in medicine as antibiotics and chemotherapy agents. It is unclear how topoisomerase inhibitors might regulate PPI in *nf1* mutants. However, topoisomerase inhibitors can activate epigenetically silenced ubiquitin protein ligase alleles in neurons associated with Angelman syndrome (Huang et al., 2011), and mouse models for Angelman syndrome display deficits in acoustic startle and PPI (Huang et al., 2013). Further research will be required to understand how these effects might relate to neurofibromin.

Overall, we identify Cxcr4 chemokine receptor signaling as a regulator of habituation and modulator of cAMP-PKA signaling in *nf1* mutant larval zebrafish, suggesting that Cxcr4 signaling may regulate neurofibromin-dependent cognitive function in addition to its roles in tumorigenesis. Clinically, plerixafor is known to mobilize stem cells from the bone marrow to the blood stream and has originally been approved by the United States Food and Drug Administration (FDA) in 2008 for autologous transplantation in patients with Non-Hodgkin's Lymphoma or multiple myeloma (De Clercq, 2019). Since then, research with plerixafor has highlighted its potential as a treatment option for various cancers (De Clercq, 2019), including the treatment of NF1-associated malignant peripheral nerve sheath tumors (Mo et al., 2013). Our work here highlights the potential for more preclinical investigation into the relationship between CXCL12-CXCR4 signaling and neurofibromin-dependent cognitive functions. Further, if CXCR4 inhibitor treatment advances toward clinical trials to treat tumors in patients with neurofibromatosis type 1, cognitive function could be considered as an additional clinical outcome.

## MATERIALS AND METHODS

### Zebrafish maintenance and breeding

Zebrafish (*Danio rerio*) larvae used in this study were generated from crosses of wild-type (Tüpfel long fin background) adults or *nf1* mutant adults carrying a combination of *nf1a^Δ5^* (*nf1a^−^*) and *nf1b^+10^* (*nf1b^−^*) mutant alleles (Shin et al., 2012). Crossing *nf1a^+/−^*;*nf1b^−/−^* and *nf1a^−/−^*;*nf1b^+/−^* adults yielded embryos with *nf1a^+/−^*;*nf1b^+/−^*, *nf1a^+/−^*;*nf1b^−/−^*, *nf1a^−/−^*;*nf1b^+/−^* or *nf1a^−/−^*;*nf1b^−/−^* mutant alleles. Genotyping of *nf1a^Δ5^* and *nf1b^+10^* alleles was performed as previously described (Shin et al., 2012). In indicated experiments, *nf1* mutant larvae were classified by the presence of a hyperpigmentation phenotype in *nf1a^−/−^*;*nf1b^−/−^* larvae. The resulting two groups of *nf1* mutant larvae are putative *nf1* heterozygous (*nf1a^+/−^*;*nf1b^+/−^*, *nf1a^+/−^*;*nf1b^−/−^*, *nf1a^−/−^*;*nf1b^+/−^*) and putative *nf1* double homozygous (*nf1a^−/−^*;*nf1b^−/−^*) mutants. This method of identification was validated by *post hoc* genotyping (100% positive predictive value and 93% negative predictive value for *nf1a^−/−^*;*nf1b^−/−^*; *n*=94).

Embryos and larvae were raised at 29°C in E3 medium (5 mM NaCl, 0.17 mM KCl, 0.33 mM CaCl2, 0.33 mM MgSO4 adjusted to pH 6.8–6.9 with NaHCO_3_) on a 14/10 h light/dark cycle. E3 medium was changed at 48 and 96 h post fertilization (hpf). Before behavior testing, larvae were maintained on a white light box (800 μW cm^−2^) for at least 1 h before being transferred to the testing arena. All experiments were conducted between 5- and 6-days post fertilization (dpf), which is before sex determination. All animals in these studies were handled in accordance with the National Institutes of Health Guide for the care and use of laboratory animals, and the University of Wisconsin Institutional Animal Care and Use Committee (IACUC). These studies were approved by the University of Wisconsin IACUC (protocol L005704).

### Behavioral recording and tracking

Behavioral responses to acoustically evoked startles were recorded as previously described (Burgess and Granato, 2007a; Wolman et al., 2011). Briefly, larvae housed individually in acrylic grids were exposed to acoustic/vibrational stimuli with a small vibration exciter (4810; Brüel and Kjaer, Norcross, GA, USA) controlled by a digital–analog card (PCI-6221; National Instruments, Austin, TX, USA). Larvae were illuminated from above with LED lights (MCWHL5 6500K LED, powered by LEDD1B driver, Thorlabs, NJ, USA) and below with infrared lights (IR Illuminator CM-IR200B, C&M Vision Technologies, Houston, TX, USA). Larval movement was recorded using MotionPro Y4 high-speed video cameras (Integrated Design Tools, Pasadena, CA, USA) with 50 mm macro lenses (Sigma Corporation of America, Ronkonkoma, NY, USA) at 1000 frames per second at either 512×512 or 1024×1024 pixel resolution.

Video images were analyzed for movement initiations and kinematics with the open-source Flote (v-2.0; https://www.nichd.nih.gov/about/org/dir/affinity-groups/AMHD/burgess/software/flote-downloads) software package as previously described (Burgess and Granato, 2007a,b; Wolman et al., 2011). Larvae that failed to initiate any movement to 60% or more of stimuli were excluded. Data from individual larvae were matched to genotype information using a custom R script that is available upon request.

### Drug screen behavioral assay

For the drug screen behavioral assay, *nf1* mutant larvae (*nf1a^+/−^*;*nf1b^+/−^*, *nf1a^+/−^*;*nf1b^−/−^*, *nf1a^−/−^*;*nf1b^+/−^*, or *nf1a^−/−^*;*nf1b^−/−^* mutant alleles, expected ratio 25% each) were tested at 5 dpf. A total of 60 acoustic stimuli were delivered with varying intensities and interstimulus intervals (ISIs) to assess baseline startle initiation, prepulse inhibition (PPI), and habituation (Fig. 1A). During the PPI phase (stimuli 1-30), a series of low-intensity, high-intensity and paired low-high-intensity acoustic stimuli were delivered, separated by 20 s ISIs, with each stimulus repeated ten times. The paired low-high-intensity stimuli were separated by 400 ms. During the proceeding habituation phase (stimuli 31-60), 30 high-intensity acoustic stimuli were delivered, separated by 1.5 s ISIs.

Baseline startle initiation probability was assessed by measuring initiations (total turns or SLCs, separately) to both low- and high-intensity stimuli during the PPI phase (stimuli 1, 2, 4, 5, 7, 8, 10, 11, 13, 14, 16, 17, 19, 20, 22, 23, 25, 26, 28, 29). The number of response initiations divided by the total analyzed was reported as the turn or SLC initiation percentage.

Habituation was assessed by calculating two ratios of SLC response initiation. The first was a ratio of SLC initiation in response to habituation phase stimuli 41-50 over SLC initiation in response to the first 10 high-intensity stimuli (PPI phase stimuli 2, 5, 8, 11, 14, 17, 20, 23, 26, 29). The second was a ratio of SLC initiation in response to stimuli 51-60 over SLC initiation in response to the first 10 high-intensity stimuli (PPI phase stimuli 2, 5, 8, 11, 14, 17, 20, 23, 26, 29). The habituation percentage is reported as 1 minus these ratios.

PPI was assessed by calculating the ratio of SLC initiations in response to paired low-high-intensity stimuli (PPI phase stimuli 3, 6, 9, 12, 15, 18, 21, 24, 27, 30) over SLC initiations in response to unpaired high-intensity stimuli (PPI phase stimuli 2, 5, 8, 11, 14, 17, 20, 23, 26, 29). The PPI percentage is reported as 1 minus this ratio.

### Habituation behavioral assay

The habituation behavioral assay was designed to be sensitive to more modest habituation deficits than the drug screen behavioral assay. Wild-type or *nf1* mutant larvae (genotyped *post hoc*) were tested in this assay at 5 dpf. A total of 50 acoustic stimuli were delivered with varying intensities and ISIs to assess baseline startle initiation and habituation (Fig. 2A). During the pre-habituation phase (stimuli 1-20), ten low-intensity stimuli were delivered, followed by ten high-intensity stimuli, all separated by 30 s ISIs. During the proceeding habituation phase (stimuli 21-50), 30 high-intensity stimuli were delivered, separated by 3 s ISIs.

Baseline startle initiation probability was assessed by measuring initiations (total turns or SLCs, separately) to both low and high-intensity stimuli during the pre-habituation phase (stimuli 1-20). The number of response initiations divided by the total analyzed was reported as the startle initiation percentage.

Habituation was assessed by calculating the ratio of SLC initiations in response to the final ten high-intensity stimuli (habituation phase stimuli 41-50) over SLC initiations in response to the first ten high-intensity stimuli (pre-habituation phase stimuli 11-20). The habituation percentage is reported as 1 minus this ratio.

### Pharmacology

For the small-molecule screen, 1134 bioactive compounds from an FDA-approved small-molecule library (Selleck) were tested on *nf1* mutant larvae. Compounds were maintained at −80°C dissolved in DMSO and were administered to larvae at a final concentration of 1 µM or 10 µM per compound (in 1% DMSO in E3 medium) for a treatment duration of 1 h before behavioral testing. Each compound was administered to 32 larvae (mix of *nf1* mutant genotypes). Two groups of 32 larvae (mix of *nf1* mutant genotypes) were left untreated on each experimental day. Behavioral testing was completed over the course of 60 experimental days.

For follow-up habituation experiments, two formulations of the Cxcr4 inhibitor AMD3100 (plerixafor 8HCl and plerixafor; Selleck) were tested on wild-type and *nf1* mutant larvae (genotyped *post hoc*). Both formulations were dissolved in water and administered to larvae in a series of doses for a treatment duration of 1 h before behavioral testing. For plerixafor 8HCl, the final concentrations in E3 medium were 300 nM, 1 µM, and 3 µM. For plerixafor, the final concentrations in E3 medium were 1 µM, 3 µM, and 10 µM. For remaining experiments (measures of ERK1/2, cAMP, and PKA signaling), the 3 µM plerixafor dose was used as treatment for a duration of 1 h.

### RNA sequencing and differential gene expression analysis

RNA was isolated from groups of 20 whole-larvae at 5 dpf using TRIzol Reagent (Invitrogen) following the manufacturer's instructions. Eight biological replicates for wild-type (Tüpfel long fin background) and putative *nf1* double mutants were processed for RNA sequencing. RNA sequencing was carried out by the Biotechnology Center Gene Expression Center at the University of Wisconsin-Madison on an Illumina HiSeq 2500 platform. Transcriptomic data processing adhered to ENCODE Consortium guidelines and best practices for RNA-Seq (ENCODE Project Consortium, 2016). Raw strand-specific paired-end (2×150 bp) Illumina reads were adapter-trimmed using Skewer (v0.1.123; Jiang et al., 2014; https://sourceforge.net/projects/skewer). Trimmed reads were aligned to the *Danio rerio GRCz11* reference genome (NCBI Assembly: GCA_000002035.4) using STAR (v2.5.3a; Dobin et al., 2013; https://github.com/alexdobin/STAR), a splice-aware aligner, with gene annotation from Ensembl release 102 (https://useast.ensembl.org/index.html). Gene- and isoform-level expression quantification was performed with RSEM (v1.3.1; Li and Dewey, 2011; https://github.com/deweylab/RSEM), which estimates transcript abundance using expectation-maximization. For downstream differential gene expression analysis, expected read counts from RSEM were rounded to the nearest integer and imported into edgeR (v3.28.0; Robinson et al., 2010; https://bioconductor.org/packages/release/bioc/html/edgeR.html), run within the R statistical environment (v3.6.2; https://www.r-project.org) Library size normalization across samples was performed using the trimmed mean of M-values (TMM) method (Robinson and Oshlack, 2010). Genes were retained for statistical testing if they exceeded a count-per-million (CPM) threshold in a minimum number of samples, defined by the number of biological replicates per condition, with a minimum read count of ten. This independent filtering step reduced noise and increased statistical power. Differential expression testing was conducted using negative binomial generalized linear models. Resulting *P*-values were adjusted for multiple testing using the Benjamini–Hochberg false discovery rate (FDR) procedure, with a significance threshold of 5% (Reiner et al., 2003). The validity of the multiple testing correction was assessed by visual inspection of the distribution of unadjusted *P*-values. KEGG pathway analysis was performed as described by Subhash and Kanduri (2016).

### Immunohistochemistry and confocal imaging

Immunostaining and whole-brain imaging for total-ERK1/2 (tERK) and phosphorylated-ERK1/2 (pERK) was performed as previously described (Randlett et al., 2015). Larvae at 6 dpf were fixed in 4% paraformaldehyde (diluted to 4% w/v in PBS from 16% w/v in 0.1 M phosphate buffer, 0.25% v/v Triton X-100 pH 7.4) overnight at 4°C. The next day, larvae were incubated in bleaching solution (3% v/v hydrogen peroxide, 1% w/v KOH, in water) for 30 min to remove pigment. Larvae were then incubated in 150 mM Tris HCl pH 9.0 for 15 min at 70°C. Larvae were permeabilized in 0.05% Trypsin-EDTA on ice for 45 min. Larvae were then incubated for 1 h in block solution (1% w/v bovine serum albumin, 2% v/v normal goat serum, 0.25% v/v Triton X-100, 1% v/v DMSO, in PBS pH 7.4). Larvae were then incubated in primary antibodies at 1:300 in incubation buffer (IB; 1% w/v bovine serum albumin, 0.25% v/v Triton X-100, 1% v/v DMSO, in PBS pH 7.4) overnight at 4°C. The next day, larvae were incubated in fluorescently conjugated secondary antibodies at 1:500 in IB overnight at 4°C and stored in Vectashield mounting medium (Vector Laboratories) until mounting for imaging. Primary antibodies included phospho-ERK1/2 antibody (Cell Signaling, #4370) and total-ERK1/2 antibody (Cell Signaling, #4696). Secondary antibodies included Alexa Fluor 488-conjugated goat anti-mouse IgG1 and Alexa Fluor 594-conjugated goat anti-rabbit IgG (Thermo Fisher Scientific, #A-21121, #A-11008) Whole-brain image stacks were acquired on an Olympus FLUOVIEW confocal laser scanning microscope (FV1000) using a 20× oil immersion objective and Fluoview software (FV10-ASW 4.2; https://evidentscientific.com/en/downloads). Images were acquired at a x/y/z resolution of 0.795/0.795/2 μM. The entire brain was captured by imaging in two sections and stitching images using the Pairwise Stitching plugin in FIJI (Preibisch et al., 2009). Pixel saturation was adjusted to 0.1% of the most intense pixels for each sample using a custom FIJI macro available upon request.

Whole-brain image stacks for total-ERK1/2 (tERK) and phosphorylated-ERK1/2 (pERK) were registered to the open-source Z-BRAIN atlas using the Jefferis lab graphical user interface for the Computational Morphometry Toolkit (CMTK) hosted on GitHub. Parameters used included: -awr 0102 -X 52 -C 8 -G 80 -R 3 -A ‘--accuracy 0.4’ -W ‘--accuracy 1.6’. Images were downsampled and smoothed using the PrepareStacksForMAPMapping FIJI macro. MAP-maps were created using the MakeTheMAPMap MATLAB script. Z-BRAIN analysis of MAP-maps was generated using the ZbrainAnalysisOfMAPMaps. FIJI and MATLAB scripts available from the open-source zebrafish atlas Z-BRAIN (https://zebrafishexplorer.zib.de/home/) hosted by the Zuse Institut Berlin (ZIB), Berlin, Germany.

Immunostaining and whole-brain imaging was performed on dissected brains for phosphorylated-PKA substrates (pPKA-substrates). Larvae at 5 dpf were fixed in 4% paraformaldehyde (diluted to 4% w/v in PBS from 16% w/v in 0.1 M phosphate buffer, 5% w/v sucrose pH 7.4) for 1 h at room temperature. Brains were dissected from the head using fine tungsten dissecting needles. Brains were permeabilized in collagenase (0.1% w/v in PBS) for 1 h and blocked for 1 h at room temperature in incubation buffer (IB; 0.2% w/v bovine serum albumin, 2% v/v normal goat serum, 0.8% v/v Triton X-100, 1% v/v DMSO, in PBS pH 7.4). Brains were incubated in primary antibodies at 1:300 in IB overnight at 4°C. Brains were incubated in fluorescently conjugated secondary antibodies at 1:500 in IB overnight at 4°C and stored in Vectashield mounting medium (Vector Laboratories) until mounting for imaging. Primary antibodies against phospho-(Ser/Thr) PKA substrate (Cell Signaling, #9621) and against total-ERK1/2 (Cell Signaling, #4696) were used. Secondary antibodies included Alexa Fluor 488-conjugated goat anti-mouse IgG1 and Alexa Fluor 594-conjugated goat anti-rabbit IgG (Thermo Fisher Scientific, #A-21121, #A-11008). Whole-brain image stacks were acquired on an Olympus Fluoview confocal laser scanning microscope (FV1000) using a 20× oil immersion objective and Fluoview software (FV10-ASW 4.2; https://evidentscientific.com/en/downloads). Images were acquired at a x/y/z resolution of 0.795/0.795/2 μM.

Whole-brain image stacks for pPKA-substrates were processed using the open-source software FIJI (v-1.53c) (Schindelin et al., 2012). Whole-brain pPKA-substrates fluorescence was measured by drawing a region of interest (ROI) around the entire brain from Z-stack average intensity projections. Background fluorescence was measured by drawing a ROI away from sample in the same projection. The total corrected fluorescence was quantified using the following calculation: integrated density−(area of ROI×mean fluorescence of the background). Fluorescence was reported as total corrected fluorescence per total brain area.

### cAMP enzyme-linked immunosorbent assay (ELISA)

For cAMP quantification, zebrafish larvae at 5 dpf were snap-frozen in liquid nitrogen. Tissue from 15 larvae per group was lysed by homogenizing in 200 µl of 0.1 M HCl using a hand-held homogenizer. Samples were spun at ∼10,000 ***g*** for 15 min at 4°C. The supernatant was transferred to a new microcentrifuge tube and diluted 1:1 in 0.1 M HCl. cAMP concentration was quantified using a direct cAMP ELISA kit (Enzo Life Science, ADI900066) following the acetylated version of the manufacturer's instructions. Three technical replicates were averaged for each sample reported.

### Statistical analysis

Descriptive statistics (mean, standard deviation, standard error) and *z*-score were calculated in the software environments RStudio (v-1.3; https://posit.co/download/rstudio-desktop/), GraphPad Prism (v-9.1.2, GraphPad Software Incorporated; https://www.graphpad.com), and MATLAB (v-9.6.0, R2019A, The MathWorks Inc.; https://www.mathworks.com/products/matlab.html). The *z*-scores for the effects of individual compounds in the small-molecule screen were calculated with the following formula: *z*-score=(mean treated value − mean non-treated control value)/(standard deviation non-treated control value). The *z*-scores were identified as significant by setting a critical value based on the one-tailed 99% confidence interval. Enrichment analysis for target compounds was performed using Fisher's exact test. Assumptions of normality were tested by Shapiro-Wilk's test. One-way ANOVA, two-way ANOVA, and Tukey's and Dunnet's multiple comparisons testing were performed with a significance level of 0.05. Gene expression analysis was performed using R (v-3.6.2; https://www.r-project.org). Data are presented as the mean±standard error of the mean (±s.e.m.). Sample sizes and statistical tests for specific experiments are identified in the figures and figure legends.

## Supplementary Material

10.1242/dmm.052509_sup1 Supplementary information
