# Supplementary material for: Inhibition of Cxcr4 chemokine receptor signaling improves habituation learning in a zebrafish model of neurofibromatosis
Source: Dis Model Mech. 2026 Mar 12;19(3):dmm052509. doi: 10.1242/dmm.052509 (PMC13035064; doi:10.1242/dmm.052509)
Supplement: Supplementary information [file dmm-19-052509-s1.pdf]

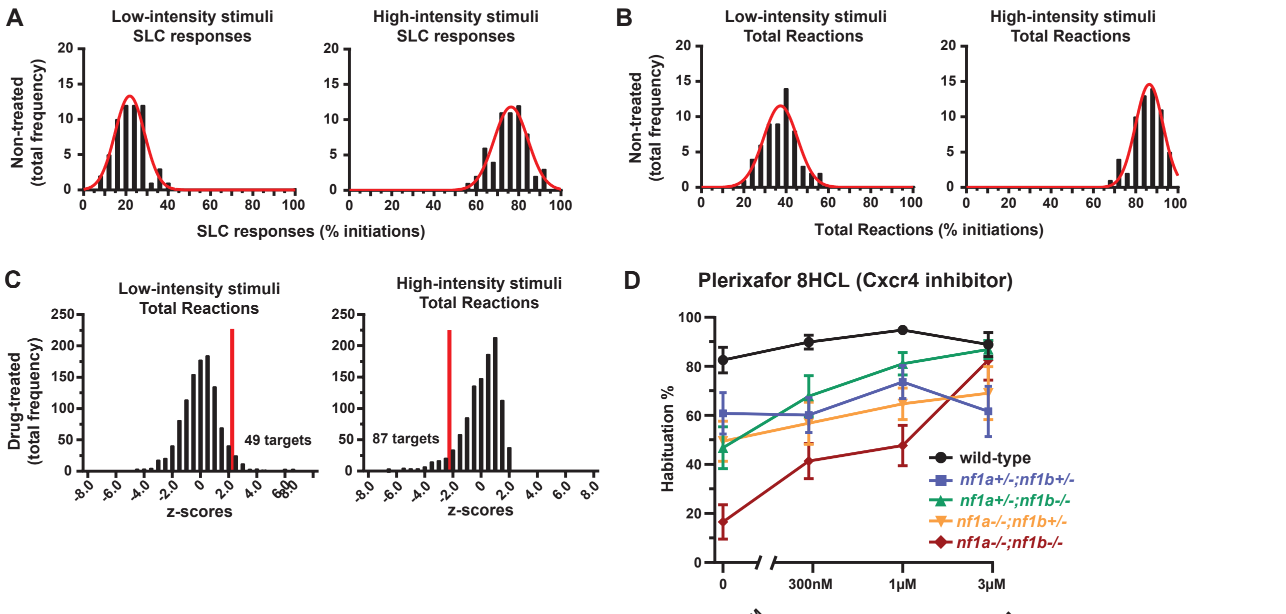

	300nM	1µM	3µM
wild-type	ns	ns	ns
nf1a+/- nf1b+/-	ns	ns	ns
nf1a+/- nf1b-/-	ns	<0.01	<0.01
nf1a-/- nf1b+/-	ns	ns	ns
nf1a-/- nf1b-/-	<0.01	<0.01	<0.01

Comparisons to non-treated larvae

	0nM	300nM	1µM	3µM
nf1a+/- nf1b+/-	0.02	<0.01	ns	ns
nf1a+/- nf1b-/-	<0.01	ns	ns	ns
nf1a-/- nf1b+/-	<0.01	<0.01	<0.01	ns
nf1a-/- nf1b-/-	<0.01	<0.01	<0.01	ns

Comparisons to wild-type larvae

Fig. S1.

(A-B) Histograms demonstrating the frequency distribution of (A) SLC responses and (B) total reactions to both low and high-intensity stimuli averaged across experimental days in 5 dpf non-treated *nf1* mutants. Each average was recorded from groups of 32 *nf1* mutant larvae. A nonlinear regression was fit to each distribution. The distribution average and standard deviation was used to calculate Z-scores to determine compounds that significantly affect behavioral measures. **(C)** Histograms demonstrating the frequency distribution of Z-scores calculated for effects of individual compounds on total reactions to both low and high-intensity stimuli. A **Z-**score threshold (2.326) was set representing the one-sided 99% confidence interval. The number of compounds identified are indicated on the graphs. Each compound was tested on a group

Disease Models & Mechanisms • Supplementary information

of 32 *nf1* mutant larvae. **(D)** Habituation ± s.e.m. for 5 dpf wild-type and *nf1* mutant larvae treated with Plerixafor 8 HCl. Data points represent average habituation from all larvae tested within each genotype at each treatment dose (sample sizes: WT control n=29, 300 nM n=26, 1 μM n=27, 3 μM n=18; *nf1a* $^{+/-}$;*nf1b*$^{+/-}$ control n=22, 300 nM n=14, 1 μM n=15, 3 μM n=9; *nf1a*$^{+/-}$;*nf1b*$^{-/-}$ control n=17, 300 nM n=14, 1 μM n=14, 3 μM n=18; *nf1a*$^{-/-}$;*nf1b*$^{+/-}$ control n=16, 300 nM n=16, 1 μM n=16, 3 μM n=9; *nf1a*$^{-/-}$;*nf1b*$^{-/-}$ control n=22, 300 nM n=24, 1 μM n=16, 3 μM n=9). Two-way ANOVA showed statistically significant differences between treatment groups ($F(3,331) = 13.90$, $p<0.001$), as well as a statistically significant interaction between genotype and treatment factors ($F(12,331) = 2.260$, $p = 0.009$). Dunnet's adjusted p-values (below graphs) were used for comparing non-treated larvae within each genotype and comparisons to wild-type larvae within each treatment dose.

Disease Models & Mechanisms • Supplementary information

A

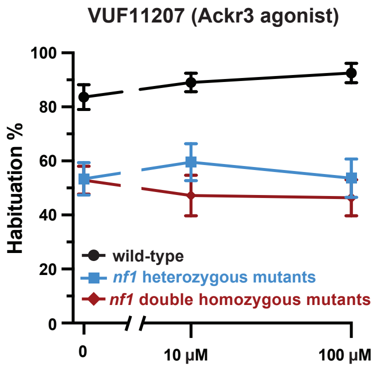

	10 µM	100 µM
wild-type	ns	ns
nf1 heterozygous	ns	ns
nf1 double homozygous	ns	ns

Comparisons to non-treated larvae

	0 µM	10 µM	100 µM
nf1 heterozygous	0.02	0.03	0.01
nf1 double homozygous	0.02	<0.01	<0.01

Comparisons to wild-type larvae

Fig. S2.

(A) Habituation ± s.e.m. for 5 dpf wild-type and *nf1* mutant larvae treated with the Ackr3 agonist VUF11207. Data points represent average habituation from all larvae tested within each group at each treatment dose (sample sizes: WT control n=8, 10 µM n=9, 100 µM n=7; *nf1* heterozygous mutants control n=31, 10 µM n=17, 100 µM n=13; *nf1* double homozygous mutants control n=36, 10 µM n=16, 100 µM n=17). Two-way ANOVA showed a statistically significant difference between genotypes ($F(2,145) = 16.94$, $p<0.001$) but no effect of treatment ($F(2,145) = 0.0513$, $p = 0.9483$). Tukey's adjusted p-values (below graphs) were used for comparing non-treated larvae within each genotype and for comparisons to wild-type larvae within each treatment dose.

Disease Models & Mechanisms • Supplementary information

A **Plerixafor 8HCL (Cxcr4 inhibitor)**

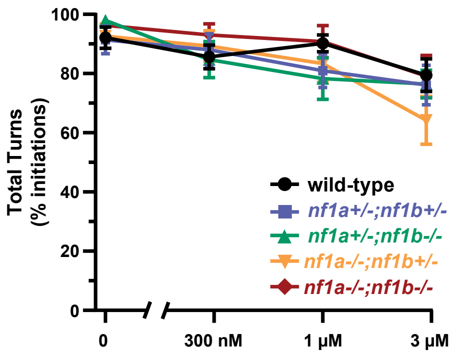

	300 nM	1 µM	3 µM
wild-type	ns	ns	ns
nf1a+/- *nf1b+/-*	ns	ns	ns
nf1a+/- *nf1b-/-*	ns	0.03	<0.01
nf1a-/- *nf1b+/-*	ns	ns	<0.01
nf1a-/- *nf1b-/-*	ns	ns	ns

Comparisons to non-treated larvae

Fig. S3.

(A) Percent initiation of total reactions ± s.e.m. in 5 dpf wild-type and *nf1* mutant larvae treated with Plerixafor 8HCl. Data points represent average initiation percentage from all larvae tested within each genotype at each treatment dose (sample sizes: WT control n=32, 300 nM n=32, 1 µM n=32, 3 µM n=30; *nf1a*$^{+/-}$*;nf1b*$^{+/-}$ control n=24, 300 nM n=17, 1 µM n=21, 3 µM n=17; *nf1a*$^{+/-}$ *;nf1b*$^{-/-}$ control n=17, 300 nM n=17, 1 µM n=18, 3 µM n=26; *nf1a*$^{-/-}$*;nf1b*$^{+/-}$ control n=17, 300 nM n=17, 1 µM n=21, 3 µM n=17; *nf1a*$^{-/-}$*;nf1b*$^{-/-}$ control n=22, 300 nM n=26, 1 µM n=20, 3 µM n=11). Two-way ANOVA showed statistically significant differences between treatment groups ($F(3,414) = 11.27$, $p<0.001$. Dunnet's adjusted p-values (below graphs) were used for comparisons between non-treated larvae within each genotype

Disease Models & Mechanisms • Supplementary information

Table S1.

Individual compounds that regulate acoustically evoked behaviors in *nf1* mutant larvae

List of small-molecule compounds identified as targets in Figure 1. Compound names and Biological Targets were defined by the manufacturer (Selleck). Z-scores that surpassed the one-sided 99% confidence interval are highlighted in the respective columns.

Compound	Biological Target	Low-intensity Total Reaction (z-score)	High-intensity Total Reaction (z-score)	Habituation stimuli 41-50 (z-score)	Habituation stimuli 51-60 (z-score)	Prepulse Inhibition (z-score)
Acebutolol HCl	Adrenergic Receptor	0.421308	0.281543	1.915521	1.366191	2.558511
Acitretin	Retinoid Receptor	3.978182	-2.36597	-11.4933	-7.72817	-17.8239
Aclidinium Bromide	AChR	-0.94632	-0.69273	0.988253	2.835919	1.523516
Allopurinol (Zyloprim)	ROS	-1.68461	-1.30177	1.761356	2.651828	0.72008
Alprostadil (Caverject)	Immunology & Inflammation related	-4.29393	-10.1228	2.495002	2.176356	3.696938
Alverine Citrate	OX Receptor	-2.03173	-3.70241	-0.38296	-0.15575	0.954885
Amantadine hydrochloride (Symmetrel)	Dopamine Receptor	2.826292	1.227472	-1.27919	-0.25266	0.271315
Amfenac Sodium (monohydrate)	COX	2.537266	1.302105	-0.34468	0.687782	-0.35063
AMG-073 HCl (Cinacalcet hydrochloride)	Others	6.214039	2.188712	-3.66821	-2.3552	-4.20904
Amiloride hydrochloride (Midamor)	Others	2.478555	1.265751	-0.70448	-0.02291	-0.2993
Amitriptyline HCl	5-HT Receptor	-2.74438	-7.09409	2.631288	0.491604	3.20173
Amorolfine Hydrochloride	Anti-infection	-2.30251	-4.59723	2.367701	2.341202	0.605162
Amoxapine	GlyT	0.632751	1.260477	2.543958	0.981392	0.847341
Ampiroxicam	COX	0.603552	0.095663	0.807294	1.152838	2.613325
Arecoline	AChR	3.01266	2.015518	0.333387	0.437928	1.057253
Aripiprazole (Abilify)	5-HT Receptor	0.020134	0.282315	1.338088	2.433811	0.992232
Asenapine	Others	3.021246	1.88583	-2.45118	-0.95165	-1.86042
Atomoxetine HCl	5-HT Receptor	-3.99261	-6.39459	2.160169	2.605673	3.294274
Atorvastatin calcium (Lipitor)	HMG-CoA Reductase	-0.09052	-0.89511	0.761225	1.197723	2.47769
Atracurium besylate	AChR	-2.41247	-4.53948	-1.89733	1.262034	0.486342
Azacitidine (Vidaza)	DNA Methyltransferase	-0.64421	-3.26243	0.062856	-0.66749	-0.27474
Azelnidipine	Calcium Channel	-2.07436	-2.76495	0.722604	1.510537	1.522512

Compound	Biological Target	Low-intensity Total Reaction (z-score)	High-intensity Total Reaction (z-score)	Habituation stimuli 41-50 (z-score)	Habituation stimuli 51-60 (z-score)	Prepulse Inhibition (z-score)
Azilsartan Medoxomil (TAK-491)	RAAS	-1.93226	-2.77738	0.061098	0.577246	1.395656
Azlocillin sodium salt	Anti-infection	-2.00674	-2.70673	-0.51041	0.51255	2.255144
Benidipine hydrochloride	Calcium Channel	-2.67413	-3.95266	1.166776	1.236766	1.358876
Benzbromarone	P450 (e.g. CYP17)	-3.01591	-5.26609	1.792406	2.24619	0.145903
Benzocaine	Sodium Channel	2.366598	1.35541	0.546085	0.086273	0.767483
Benztropine mesylate	Dopamine Receptor	-0.3416	0.213576	2.607055	1.907421	1.072365
Besifloxacin HCl (Besivance)	Anti-infection	-1.87377	-2.76312	2.032134	1.928163	2.388905
Betamethasone (Celestone)	Glucocorticoid Receptor	-0.07529	-0.31518	-0.2989	0.499749	2.653669
BIBR 953 (Dabigatran etexilate, Pradaxa)	Anti-infection	-1.90987	-3.18777	0.037313	0.187195	1.568575
Bifonazole	Anti-infection	0.652738	-3.90904	2.608574	2.644551	-1.16206
Bleomycin sulfate	DNA/RNA Synthesis	-1.3496	-2.48893	0.408625	0.694112	1.023436
Bumetanide	Others	-1.55914	-2.40896	0.133825	-0.12731	-0.43519
Calcitriol (Rocaltrol)	Vitamin	-3.11756	-5.47654	0.812808	1.21524	-0.39294
Carvedilol	DNA/RNA Synthesis	-1.88249	-2.62676	-1.81935	2.124067	1.087218
Caspofungin acetate	DNA/RNA Synthesis	-2.81052	-3.95944	-1.82237	-1.65394	1.1341
Celecoxib	COX	-2.94081	-3.75779	2.150513	2.191325	-0.71837
Chloramphenicol (Chloromycetin)	Anti-infection	2.384046	1.295524	1.296416	1.139134	1.692065
Chlorocresol	Calcium Channel	2.333651	1.057426	-1.65341	-0.91732	0.050184
Chloroxine	Anti-infection	2.899993	1.74467	1.205407	2.112354	-2.64596
Chlorpromazine (Sonazine)	Potassium Channel, Dopamine Receptor	-1.82025	-0.28737	1.820779	3.118333	1.506288
Chlorpropamide	Others	-0.78162	-0.55512	1.556138	1.531455	2.540194
Chlortetracycline HCl	Anti-infection	-0.00549	-0.65544	1.155105	2.328873	1.20366
Chlorzoxazone	P450 (e.g. CYP17)	-0.73525	-0.80768	2.320023	2.471749	1.489087
Cilnidipine	Calcium Channel	-2.63704	-7.11322	-2.21013	0.576538	2.349453
Cleviprex (Clevidipine)	Calcium Channel	0.0789	0.010525	1.593188	2.395967	2.125829
Clomipramine hydrochloride (Anafranil)	5-HT Receptor	-2.97173	-3.15071	1.435527	1.895656	0.866145
Closantel	Anti-infection	-3.04217	-6.23522	1.975814	3.086274	3.014535
Crystal violet	Others	-4.10235	-6.32222	-13.2203	-9.13011	-0.38262

Disease Models & Mechanisms • Supplementary information

Compound	Biological Target	Low-intensity Total Reaction (z-score)	High-intensity Total Reaction (z-score)	Habituation stimuli 41-50 (z-score)	Habituation stimuli 51-60 (z-score)	Prepulse Inhibition (z-score)
Deferasirox (Exjade)	P450 (e.g. CYP17)	-3.09852	-5.01799	-0.04602	0.327068	1.466917
Deoxycorticosterone acetate	Adrenergic Receptor	2.52218	1.834077	0.544899	1.066751	-1.62806
Detomidine HCl	Adrenergic Receptor	-1.3203	-2.62053	2.455064	2.773297	1.137442
Dexmedetomidine HCl (Precedex)	Adrenergic Receptor	0.96478	0.130373	2.160723	2.512874	1.758576
Dextrose (D-glucose)	NA	2.619867	1.745691	0.483317	0.248384	0.174683
Dibenzothiophene	NA	2.618149	0.800315	-0.22881	-0.17572	-1.45731
Dichlorisone Acetate	NA	3.039094	2.01688	-1.71742	-1.17535	-1.29533
Dicyclomine HCl	Others	-1.93242	-2.43647	-0.32131	-0.09384	-0.35077
Diethylstilbestrol (Stilbestrol)	Estrogen / progestogen Receptor	-3.44885	-9.20853	1.28511	3.434416	-0.27641
Difluprednate	Others	1.073041	-0.41123	2.291753	1.96536	2.609367
Diperodon HCl	Others	2.626425	1.638508	-0.40898	-0.22617	-0.93076
Doxercalciferol (Hectorol)	Vitamin	-2.77639	-2.72601	1.548088	1.544834	0.880856
Doxofylline	PDE	-0.44988	0.690879	1.457308	2.468547	1.978805
Drospirenone	Estrogen / progestogen Receptor	-1.50385	-3.58955	1.087269	-0.41493	0.093481
Duloxetine HCl (Cymbalta)	5-HT Receptor	-1.15512	-3.57433	2.273265	0.350268	1.695556
Dutasteride	5-alpha Reductase	-2.39773	-5.01038	-0.2191	0.078269	0.901781
Dyclonine HCl	Sodium Channel	-0.16281	0.060628	2.539832	2.09776	1.587401
Enoxacin (Penetrex)	Topoisomerase	-2.33652	-2.87116	2.359797	2.072229	2.487949
Enrofloxacin	Anti-infection	-2.95031	-3.74461	0.816555	2.298714	1.806858
Epinephrine bitartrate (Adrenalinium)	Adrenergic Receptor	4.151504	1.951379	2.081548	2.349438	0.854977
Erlotinib HCl	EGFR	-2.4701	-3.72316	-2.84772	0.324386	0.514782
Escitalopram oxalate	5-HT Receptor	-0.20244	-0.26968	0.141512	0.106302	2.350175
Estradiol	Estrogen / progestogen Receptor	-4.41015	-10.4013	3.376039	2.498589	3.153063
Ethacridine lactate monohydrate	Anti-infection	-2.47063	-2.61963	-0.36537	0.279382	-0.79685
Ethoxzolamide	Others	-1.46991	-2.59072	1.301666	0.143869	0.344243
Etomidate	GABA Receptor	6.533383	2.283645	-1.83113	-1.8623	-5.77916
Etoposide (VP-16)	Topoisomerase	- 1.361860	- 2.613320	0.407693	-0.688181	1.813234
Evista (Raloxifene Hydrochloride)	mTOR	-1.96727	-3.25789	-0.34413	0.644483	0.681027
Felbamate	NMDAR	2.423903	1.464847	-3.5872	-2.71284	-2.24664

Disease Models & Mechanisms • Supplementary information

Compound	Biological Target	Low-intensity Total Reaction (z-score)	High-intensity Total Reaction (z-score)	Habituation stimuli 41-50 (z-score)	Habituation stimuli 51-60 (z-score)	Prepulse Inhibition (z-score)
Fenoprofen calcium	Immunology & Inflammation related	2.419646	1.384945	-2.54282	-3.20058	-1.31771
Finasteride	5-alpha Reductase	-1.45961	-2.87047	-1.83036	-2.24565	1.165341
Fluconazole	P450 (e.g. CYP17)	-0.08669	1.307592	2.046619	2.222052	2.814241
Flumazenil	GABA Receptor	2.512103	1.696075	-2.51948	-1.72657	-1.69189
Fluvoxamine maleate	5-HT Receptor	-1.44378	-0.70475	2.335901	2.598347	0.34965
Ftorafur	NA	-0.69939	-1.34712	0.595654	1.070845	2.501992
Furosemide (Lasix)	Sodium Channel	3.073544	1.670447	-1.80722	-1.19555	-1.0937
Gabapentin (Neurontin)	GABA Receptor	0.302814	-0.28558	2.372807	1.839923	0.569497
Genistein	EGFR, Topoisomerase	-3.37833	-6.89121	2.868168	2.956799	3.440108
Hyoscyamine (Daturine)	AChR	-0.16349	-0.65528	-0.72231	0.031917	2.328959
Ifosfamide	DNA/RNA Synthesis	-0.59951	0.095462	1.437829	1.889965	2.772529
Iloperidone (Fanapt)	5-HT Receptor	2.989621	2.071364	-1.7861	-1.25266	-0.48867
Imatinib Mesylate	Bcr-Abl, c-Kit, PDGFR	-1.74176	-0.16099	1.517681	0.979593	2.404743
Imipramine HCl	Others	-2.69516	-2.53289	2.745015	2.459705	1.554301
Imiquimod	Immunology & Inflammation related	-2.12816	-3.78363	-1.37733	-4.5849	1.073567
Irinotecan	Topoisomerase	-1.01973	-1.98945	0.505238	0.617193	2.425176
Isoxicam	NA	3.155791	2.120319	-2.59136	-2.55838	-2.3253
Isradipine (Dynacirc)	Calcium Channel	-3.92978	-8.2439	-1.02488	1.500642	2.683792
Levamisole Hydrochloride (Ergamisol)	Immunology & Inflammation related	-1.00508	-2.68452	-0.69704	-0.41508	1.502713
Levofloxacin (Levaquin)	Topoisomerase	2.44868	0.947148	-3.01474	-2.1218	-1.03826
Levonorgestrel (Levonelle)	Estrogen / progestogen Receptor	-3.44391	-5.42941	-1.02342	-1.03469	0.558819
Lomustine (CeeNU)	DNA/RNA Synthesis	-2.68463	-3.65625	-3.66974	-3.46101	0.509396
Loratadine	Histamine Receptor	6.162984	2.234647	-3.42546	-2.85107	-5.44635
Malotilate	Others	-2.1258	-2.44573	-1.78018	-2.57236	-2.0673
MDV3100 (Enzalutamide)	c-Kit, PDGFR	2.528144	1.935217	-0.94254	-0.49721	-1.03652
Medetomidine HCl	Adrenergic Receptor	-1.16295	-3.05847	2.560893	2.868119	1.855348
Mefenamic acid	COX	3.583205	1.97877	-2.93854	-2.86635	-3.12714
Methscopolamine (Pamine)	AChR	-0.83672	-0.08322	1.735141	1.497091	2.624781
Mexiletine HCl	Sodium Channel	-1.42073	-3.03525	-0.36593	0.56033	0.828205

Compound	Biological Target	Low-intensity Total Reaction (z-score)	High-intensity Total Reaction (z-score)	Habituation stimuli 41-50 (z-score)	Habituation stimuli 51-60 (z-score)	Prepulse Inhibition (z-score)
Mianserin hydrochloride	Histamine Receptor	1.098691	1.653395	2.051582	2.698842	1.337899
Miconazole nitrate	Anti-infection	-3.05605	-3.72538	2.185008	1.694363	1.790361
Monobenzone (Benoquin)	Tyrosinase	-1.42947	-2.94314	-2.80863	-1.7141	2.662652
Nafamostat mesylate	Serine Protease	-0.33005	0.392894	1.298217	2.586782	1.611949
Naftopidil (Flivas)	Adrenergic Receptor	2.810859	1.645313	0.385752	0.943468	0.889912
Netilmicin Sulfate	Anti-infection	-2.59573	-3.79752	-0.14516	1.683154	2.664478
Nilotinib (AMN-107)	Bcr-Abl	-1.88366	-3.2281	0.350868	0.857194	2.267196
Nimodipine (Nimotop)	Autophagy, Calcium Channel	-4.28226	-2.81355	3.345204	2.098739	-5.50049
Nisoldipine (Sular)	Calcium Channel	-3.64675	-7.35319	3.955216	3.561014	4.145767
Norfloxacin (Norxacin)	Topoisomerase	-3.17284	-5.03694	-0.06236	0.536054	3.137584
olsalazine sodium	Anti-infection	0.530279	0.769613	0.87366	0.549903	2.343754
Oxybutynin (Ditropan)	AChR	-3.12139	-2.5005	2.652056	2.654832	0.592397
Oxytetracycline (Terramycin)	Anti-infection	-2.46777	-2.66433	1.645679	2.268499	1.95702
Paromomycin Sulfate	Anti-infection	-0.90045	-2.75308	0.691035	0.593974	0.622171
Pazopanib	c-Kit, PDGFR, VEGFR	2.463096	0.986226	-3.06004	-2.28175	-0.84305
Penfluridol	Dopamine Receptor	0.014331	0.023139	1.255418	2.468923	2.183519
Pergolide mesylate	Dopamine Receptor	-2.09512	-2.79211	1.667547	1.126088	1.533419
Pheniramine Maleate	Histamine Receptor	-3.06354	-5.48881	1.883675	1.029556	1.240885
Phenylephrine HCl	Adrenergic Receptor	-1.21598	-0.65	1.961588	2.741951	0.991361
Piromidic Acid	Others	3.04523	1.58124	-0.78469	-0.53593	-0.19587
Piroxicam (Feldene)	COX	-2.8668	-3.54145	-3.28321	-0.87068	1.499721
Pitavastatin calcium (Livalo)	HMG-CoA Reductase	-2.12227	-4.1929	1.966497	0.600294	-0.71941
Pizotifen malate	5-HT Receptor	2.469186	1.761003	0.150826	0.451528	0.392392
Plerixafor (AMD3100)	CXCR	1.248123	0.79636	2.337142	1.670019	2.103862
Pregnenolone	Estrogen / progestogen Receptor	-1.08639	-2.60407	0.204998	-0.40631	0.112481
Procaine (Novocaine) HCl	Anti-infection	-1.08425	-0.82313	0.938866	1.39973	2.543957
Protionamide (Prothionamide)	Anti-infection	-2.39476	-3.70332	-0.69687	-0.38614	0.929215
Pyrithione zinc	Proton Pump, Anti-infection	-3.39147	-4.738	-4.10342	-2.94806	-0.49392

Disease Models & Mechanisms • Supplementary information

Compound	Biological Target	Low-intensity Total Reaction (z-score)	High-intensity Total Reaction (z-score)	Habituation stimuli 41-50 (z-score)	Habituation stimuli 51-60 (z-score)	Prepulse Inhibition (z-score)
Ractopamine HCl	Others	2.972278	2.178891	-0.77249	-1.30704	-0.50659
Reboxetine mesylate	Others	-2.37837	-3.30912	1.701515	1.775376	2.734845
Resveratrol	Autophagy	2.476726	1.983023	-0.04071	0.030355	-0.64519
Riluzole (Rilutek)	GluR, Sodium Channel	4.155877	1.551304	0.454758	-0.65313	-5.78648
Rimonabant (SR141716)	Cannabinoid Receptor	3.757165	1.96866	-4.4425	-5.22721	-4.26326
Risedronic acid (Actonel)	NA	-3.10137	-4.58469	3.284599	2.297385	1.312182
Risperidone (Risperdal)	5-HT Receptor	-2.09411	-0.26657	2.633452	2.715713	1.183495
Rivaroxaban (Xarelto)	Factor Xa	3.728505	2.236178	-0.77368	-1.45299	-1.33439
Rizatriptan Benzoate (Maxalt)	5-HT Receptor	-0.61718	0.423092	2.194615	1.410742	2.4724
Roflumilast (Daxas)	PDE	-2.09169	-3.72878	-0.54814	-2.6528	3.207306
Rolipram	PDE	-3.11713	-5.27678	1.174834	2.54847	2.568928
Rolitetracycline	PDE	2.35482	1.104839	0.834422	0.008613	1.536146
Ropivacaine HCl	PDE	-2.03501	-3.08444	-0.05032	0.931685	2.021863
Sarafloxacin HCl	Anti-infection	-1.928	-3.22107	-0.53965	1.877445	1.064599
Sertraline HCl	5-HT Receptor	-2.81542	-5.32984	1.886099	1.873202	3.69223
Sitafloxacin hydrate	Anti-infection	-2.3167	-2.33303	1.062152	1.513251	1.348962
Sodium Gluconate	Others	-0.07384	0.004862	0.02373	0.041292	2.41692
Sorafenib (Nexavar)	PDGFR, Raf, VEGFR	-3.22883	-3.7036	1.703912	1.91252	0.313793
Sotalol (Betapace)	Adrenergic Receptor	2.358418	1.487432	-2.78704	-1.96996	-3.61898
Spectinomycin hydrochloride	Anti-infection	2.395651	1.891663	-2.45555	-2.48384	-2.77414
Sulfamethizole (Proklar)	Anti-infection	-1.63699	-1.61556	1.53491	0.567233	2.478949
Sumatriptan succinate	5-HT Receptor	2.537109	1.47144	-0.91147	-1.29982	0.433707
Sunitinib Malate (Sutent)	c-Kit, PDGFR, VEGFR	-2.27656	-3.89267	-1.61475	-0.34152	0.620182
Suprofen (Profenal)	COX	-1.81203	-2.80394	0.945676	0.505011	0.59218
Tadalafil (Cialis)	PDE	-1.29705	-1.2088	1.378144	1.931921	3.136495
Tazarotene (Avage)	Retinoid Receptor	6.296766	2.148629	-4.73147	-3.96366	-5.45499
Tebipenem pivoxil (L-084)	Anti-infection	-2.02044	-2.71412	-0.76274	-0.62592	-0.38115
Terbinafine (Lamisil, Terbinex)	Anti-infection	-2.95611	-3.66054	-2.06765	-1.00166	1.720487
Terfenadine	Others	1.148227	0.91325	-0.91236	-0.0034	2.574038
tetrahydrozoline hydrochloride	Adrenergic Receptor	-2.15227	-2.89548	0.44342	1.755001	1.079063
Tianeptine sodium	5-HT Receptor	3.060531	2.08221	-1.23418	-1.61037	-0.88374
tinidazole	Anti-infection	2.337464	1.137173	-0.6419	-1.60288	0.130478
Tobramycin	Anti-infection	3.251946	1.571647	0.591653	0.458885	0.701805

Disease Models & Mechanisms • Supplementary information

Compound	Biological Target	Low-intensity Total Reaction (z-score)	High-intensity Total Reaction (z-score)	Habituation stimuli 41-50 (z-score)	Habituation stimuli 51-60 (z-score)	Prepulse Inhibition (z-score)
Tolnaftate	Anti-infection	-3.26407	-4.96362	-3.6	-3.72485	0.020652
Toremifene Citrate (Fareston, Acapodene)	Estrogen / progestogen Receptor	4.742518	0.954017	-3.638	-2.7311	-2.45292
Tranexamic acid (Transamin)	Others	-1.62993	-0.97723	2.699371	2.458322	1.487175
Triclabendazole	Microtubule Associated	-4.2646	-8.82115	-0.23189	-1.56121	-2.98622
Trilostane	Dehydrogenase	-1.99675	-3.31741	-0.73957	-3.64485	0.704744
Valproic acid sodium salt (Sodium valproate)	GABA Receptor, HDAC, Autophagy	-0.67797	-0.14443	1.940246	2.090855	2.3452
Vardenafil (Vivanza)	PDE	2.85599	1.782779	-1.61491	-1.6149	-1.57769
Varenicline tartrate	AChR	-0.27415	-0.18556	0.985315	2.58431	1.311162
Vinpocetine (Cavinton)	Sodium Channel	2.584734	0.981705	-0.2796	-0.58491	-0.02913
Vitamin D3 (Cholecalciferol)	NA	1.052784	0.541886	-0.06757	0.071739	2.752007
Xylazine HCl	Adrenergic Receptor	2.582946	0.89071	0.720803	0.350189	0.675392
Ziprasidone hydrochloride	5-HT Receptor, Dopamine Receptor	-0.56489	1.050268	1.766881	2.490617	0.833657
